# NCoR1 controls *Mycobacterium tuberculosis* growth in myeloid cells by regulating the AMPK-mTOR-TFEB axis

**DOI:** 10.1371/journal.pbio.3002231

**Published:** 2023-08-17

**Authors:** Viplov Kumar Biswas, Kaushik Sen, Abdul Ahad, Arup Ghosh, Surbhi Verma, Rashmirekha Pati, Subhasish Prusty, Sourya Prakash Nayak, Sreeparna Podder, Dhiraj Kumar, Bhawna Gupta, Sunil Kumar Raghav

**Affiliations:** 1 Immuno-genomics & Systems Biology Laboratory, Institute of Life Sciences (ILS), Bhubaneswar, India; 2 School of Biotechnology, Kalinga Institute of Industrial Technology (KIIT), Bhubaneswar, India; 3 Regional Centre for Biotechnology, Faridabad, India; 4 Molecular Medicine: Cellular Immunology, International Centre for Genetic Engineering and Biotechnology (ICGEB), New Delhi, India; The Francis Crick Institute, UNITED KINGDOM

## Abstract

*Mycobacterium tuberculosis* (*Mtb*) defends host-mediated killing by repressing the autophagolysosome machinery. For the first time, we report NCoR1 co-repressor as a crucial host factor, controlling *Mtb* growth in myeloid cells by regulating both autophagosome maturation and lysosome biogenesis. We found that the dynamic expression of NCoR1 is compromised in human peripheral blood mononuclear cells (PBMCs) during active *Mtb* infection, which is rescued upon prolonged anti-mycobacterial therapy. In addition, a loss of function in myeloid-specific NCoR1 considerably exacerbates the growth of *M*. *tuberculosis* in vitro in THP1 differentiated macrophages, ex vivo in bone marrow-derived macrophages (BMDMs), and in vivo in NCoR1^MyeKO^ mice. We showed that NCoR1 depletion controls the AMPK-mTOR-TFEB signalling axis by fine-tuning cellular adenosine triphosphate (ATP) homeostasis, which in turn changes the expression of proteins involved in autophagy and lysosomal biogenesis. Moreover, we also showed that the treatment of NCoR1 depleted cells by Rapamycin, Antimycin-A, or Metformin rescued the TFEB activity and LC3 levels, resulting in enhanced *Mtb* clearance. Similarly, expressing NCoR1 exogenously rescued the AMPK-mTOR-TFEB signalling axis and *Mtb* killing. Overall, our data revealed a central role of NCoR1 in *Mtb* pathogenesis in myeloid cells.

## Abstract 2

This is the first report depicting role of a Nuclear Receptor Co-repressor-1 (NCoR1) regulating autophagy induction and lysosomal biogenesis in myeloid cells by fine-tuning ATP homeostasis through AMPK-mTOR-TFEB signaling axis thereby controlling Mycobacterium tuberculosis pathogenesis.

## Introduction

Macrophages and dendritic cells (DCs) are the most important cells in the myeloid lineage. They have amazing phagocytic abilities that allow them to eliminate intracellular pathogens like *Mycobacterium tuberculosis* (*Mtb*) from the host [1–3]. Among them, macrophages are central for both the host and the *Mtb* pathogen, as they provide the first line of defence and also a favourable niche for the *Mtb* survival, respectively [2,4]. These immune cells possess different pathogen recognition receptors (PRRs) to detect pathogens, including *Mtb* and orchestrate diverse innate immune defence strategies such as phagocytosis, autophagy, and inflammasome activation [5]. However, *Mtb* hijacks host defence machinery by arresting phagosome maturation and rendering the local environment suitable for survival advantage [6,7]. The virulence of *Mtb* contributes to impairing the fusion of the auto-phagosome to the lysosome, i.e., autophagy flux, in order to evade the host antimicrobial mechanism [8,9]. However, the precise process by which *Mtb* inhibits particular stages of the autophagy pathway is not well understood. To establish a successful invasion, *Mtb* dismantles macrophage functionality, weakening the effectiveness of lysosomal trafficking for its own survival advantages [10]. Alveolar macrophages have been shown to be replication permissive for *Mtb*, which migrates to the interstitium to infect additional phagocytic myeloid cells in IL1β-dependent manner, suggesting a tug of war between *Mtb* and the host immune system [11]. It is still a crucial question to address how *Mtb* triggers cellular death to get dispersed in new phagocytic cells by augmenting inflammation. The transcription factor EB (TFEB) is a well-known master regulator that drives the expression of genes involved in autophagy [12]. Nutrient deprivation (starvation) studies demonstrated that de-phosphorylation of TFEB and its subsequent nuclear translocation are important for optimum autophagy induction and to retrieve energy levels by adenosine triphosphate (ATP) synthesis from internal metabolic sources [13,14]. Eukaryotic cells evolved to rewire intracellular metabolic cues to connect with molecular sensors, facilitating autophagy induction or inhibition for homeostasis maintenance. Among them, AMP-activated protein kinase (AMPK) is known as a key cellular energy sensor that undergoes phosphorylation to become activated in low energy states (low ATP levels) [15,16]. Activated AMPK then directly or indirectly suppresses mTOR activity, which is generally present on the lysosomal compartment to trap TFEB [16]. The mTOR inhibition through AMPK in a low cellular energy state leads to de-phosphorylation of TFEB and subsequent nuclear translocation to activate genes for optimum autophagy induction and lysosome biosynthesis, hence fuelling up cellular energy levels [17]. Moreover, pharmacological inhibition of AMPK and mTOR has been used to prove their key roles in autophagy flux [18,19]. There is strong evidence that *Mtb* suppresses the autophagy in cells through down-regulation of TFEB activity via the AMPK-mTOR pathway, indicating the hidden molecular network that facilitates cross-talk of these pathways under *Mtb* pathogenesis [20–23]. Hence, it is highly anticipated to identify the host factors (transcriptional modulators) that regulate autophagy flux during *Mtb* infection, which could be further harnessed to develop novel paradigms to control *Mtb* infection. Among such, nuclear receptor corepressor, NCoR1, is well known to act with multi-protein complex partners to exert repression of a wide variety of genes involved in various biological processes [24–26]. Recently, it has been shown that NCoR1 gets accumulated in autophagy-deficient mice leading to suppression of LXRα-dependent genes regulating the fatty acid synthesis and lipid droplet formation, suggesting the tight connection between NCoR1 and autophagy function [27]. NCoR1 has also been shown as a key transcriptional checkpoint for the maintenance of energy metabolism in cells, and depletion of NCoR1 leads to an increase in oxidative metabolism and ATP levels [28–30]. *Mtb* has been identified to target key metabolites to perturb intracellular energy levels to escape from host autophagic machinery [31]. Energy fluctuation caused by cellular stress directly impacts the autophagy machinery through AMPK pathway, suggesting a possible role of NCoR1 in controlling *Mtb* infection.

The primary objective of the present study is to determine NCoR1’s contribution to *Mtb* pathogenesis. We observed that NCoR1 expression is elevated in myeloid cells early in *Mtb* infection, and its loss of function impairs the auto-phagolysosome process via the AMPK-mTOR-TFEB axis, promoting its survival. Our findings unequivocally demonstrated that NCoR1 plays a crucial protective role in regulating *Mtb* clearance from host cells.

## Results

### NCoR1 expression is increased upon *Mycobacterium* infection in myeloid cells

**Fig 1:**
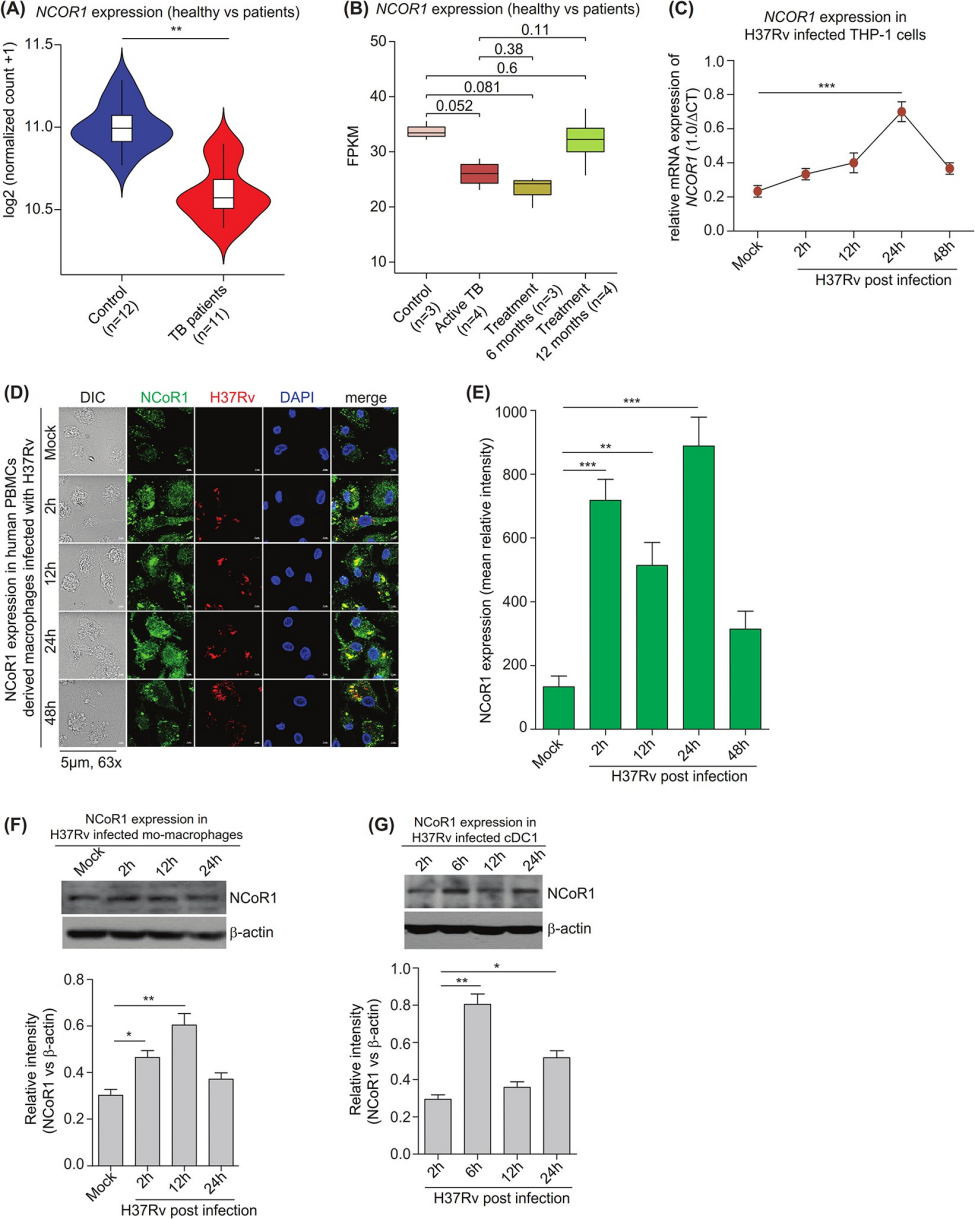
NCoR1 expression is increased upon *Mycobacterium* infection in myeloid cells. (A) Violin plot depicting the normalised read counts of *NCOR1* in publicly available transcriptome data of PBMCs from active TB patients and healthy control. Healthy control *n* = 12, active TB patients *n* = 11. (B) Box plot demonstrating the normalised FPKM (Fragment counts / kb / million reads) of *NCOR1* from publicly available transcriptome data of PBMCs from healthy control and active TB patients along with active patients undergoing anti-TB treatment regimen for 6 and 12 months. Healthy control *n* = 3, active patients *n* = 4, active patients with 6 months treatment *n* = 3, active patients with 12 months treatment *n* = 4. (C) RT-qPCR line graph depicting the *NCOR1* transcription kinetics (2 h, 6 h, 24 h, and 48 h) in H37Rv-infected human monocytic THP-1 differentiated macrophages (*n* = 3). (D, E) Confocal microscopy images and bar plots quantification showing the NCoR1 expression in human PBMCs-derived macrophages infected with H37RV at different time points (*n* = 4 human subjects). (F) Western blot image with densitometric analysis (bar plots) of bands depicting the NCoR1 protein expression upon H37Rv infection in human monocytic THP-1 differentiated macrophages at different time points. For normalisation, β-actin was used as housekeeping control (*n* = 3). (G) Western blot image and its densitometric analysis (bar plots) of bands demonstrating the NCoR1 protein levels in H37Rv-infected mouse cDC1 at different time points (*n* = 3). *p < 0.05, *p < 0.01, and ***p < 0.001 were considered significant. Data analysis was performed, (A) Wald test, (B) Wilcoxon rank sum test and others using a one-way ANOVA with Tukey’s statistical test. Where *n* represents independent biological replicates. The data underlying this figure are available in S3 and S4 Tables and S1 Data. Western blot raw images can be found in S1 Raw Image. PBMC, peripheral blood mononuclear cell; TB, tuberculosis.

Initially, we assessed the whole blood gene expression profile of patients with culture positive tuberculosis (TB) infection against healthy controls curated from the NCBI GEO database. When we compared individuals with active tuberculosis to healthy controls, we found that the expression of the *NCOR1* transcript was significantly lower in the active TB cases (**Fig 1A**) [32,33]. The same expression profile was observed in a separate RNA-seq dataset where the gene expression profile of TB-infected patients was captured during the course of antituberculosis treatment. We observed a decreased *NCOR1* transcript expression upon active TB infection and 6 months of treatment in treated subjects as compared to controls, whereas after 12 months of anti-mycobacterial therapy, the *NCOR1* expression is restored near to control levels (**Fig 1B**) [34]. These data suggested a clinical significance of NCoR1 levels in *Mtb* infection. Then, we looked into the kinetics of *NCOR1* expression in H37Rv-infected human monocytic THP-1 differentiated macrophages (mo-MΦ) using RT-qPCR. We observed dynamic changes in the *NCOR1* transcript levels, where its expression was found to be significantly increased during early time points till 24 h and then it went down at 48 h post infection (**Fig 1C**) [35]. It has been reported that the expression of early host response genes during *Mtb* infection is a crucial event to decide the fate of the disease outcome [33,36]. *Mtb* takes over the cellular regulatory circuit at later stages to subvert host defence machinery for its survival advantage [7,37,38]. To substantiate our observations further, we infected human peripheral blood mononuclear cells (PBMCs) with H37Rv to analyse NCoR1 protein levels by immunofluorescence (**Fig 1D and 1E)**. The expression of *NCOR1* in PBMCs was also confirmed at the transcript levels (**S1A Fig**). We further assessed the NCoR1 protein levels in *Mtb*-infected mo-MΦ using western blotting and found its peak expression at 12 h post infection, which goes down at later time points (**Fig 1F**). Next, we asked whether this dynamic expression of NCoR1 is limited to mo-MΦ or whether it behaves similarly in other phagocytic myeloid cell types as well, like DCs. We employed a murine-derived type 1 conventional dendritic cell line (Mutu-cDC1 line), which was characterised in detail in previous reports [39–41]. We checked the NCoR1 expression in *Mtb* and *M*. *smegmatis*-infected cDC1 line [40,42]. Though cDC1 showed early induction of NCoR1 at 6 h in western blot analysis post infection, the trend was similar to the results observed in macrophages (**Fig 1G**). In addition, *Ncor1* expression also showed a similar increased pattern at the transcript level as well as in *M*. *smegmatis*-infected cDC1 line (**S1B Fig**). Overall, these results depicted that NCoR1 expression was significantly increased during the early course of H37Rv infection, which goes down after 24 h, hinting towards an important role in host responses against *Mtb* infection.

### NCoR1 depletion enhanced *Mycobacterium* burden in myeloid cells

**Fig 2:**
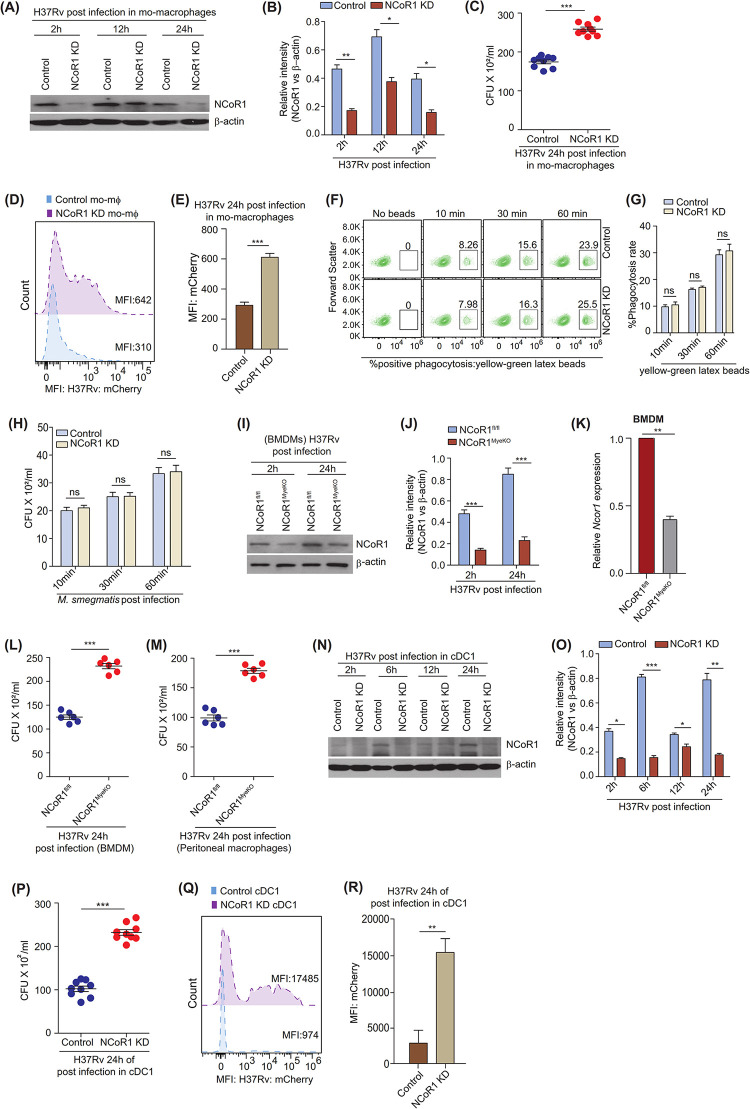
NCoR1 depletion enhanced *Mycobacterium* burden in myeloid cells. (A) Western blot image showing the NCoR1 protein levels at 2 h, 12 h, and 24 h in H37Rv-infected control and NCoR1 depleted human monocytic THP-1 differentiated macrophages. For normalisation, β-actin was used as housekeeping control (*n* = 3). (B) Bar plot demonstrating the quantification of NCoR1 protein levels at 2 h, 12 h, and 24 h of H37Rv-infected control and NCoR1 KD human monocytic THP-1 differentiated macrophages. Densitometric analysis is performed using 3 independent biological replicates (*n* = 3). (C) Scatter plot showing the bacterial load in H37Rv-infected control and NCoR1 KD human monocytic THP-1 differentiated macrophages by CFU assay 24 h post infection (*n* = 9). (D) Representative flow cytometry histogram plots showing the MFI shift of H37Rv-mCherry–infected control and NCoR1 KD human monocytic THP-1 differentiated macrophages at 24 h post infection (*n* = 3). (E) Bar plot demonstrating the quantification of MFI shifts from 3 biological replicates of H37Rv-mCherry–infected control and NCoR1 KD human monocytic THP-1 differentiated macrophages at 24 h post infection (*n* = 3). (F, G) Flow cytometry contour plot and bar plot showing the phagocytosis rate of yellow-green latex beads in control and NCoR1 KD human monocytic THP-1 differentiated macrophages at 10 min, 30 min, and 60 min post latex bead incubation (*n* = 3). (H) Bar plot showing the quantification of phagocytosis rate estimated by CFU assay of *M*. *smegmatis* in control and NCoR1 KD human monocytic THP-1 differentiated macrophages at 10 min, 30 min, and 60 min post infection (*n* = 3). (I, J) Western blot image with densitometric analysis of bands depicting the NCoR1 protein levels at 2 h and 24 h of H37Rv-infected BMDMs generated from NCoR1^fl/fl^ and NCoR1^MyeKO^ mice. For normalisation, β-actin was used as housekeeping control. Three independent biological replicates were used to estimate the protein levels (*n* = 4 mice). (K) Bar plot demonstrating the NCoR1 transcript levels in BMDMs generated from NCoR1^fl/fl^ and NCoR1^MyeKO^ mice post 24 h of H37Rv infection. Three independent biological replicates were used to estimate the transcript levels (*n* = 4 mice). (L) Scatter plot showing the bacterial load in H37Rv-mCherry–infected BMDMs generated from NCoR1^fl/fl^ and NCoR1^MyeKO^ mice (*n* = 6 mice). (M) Scatter plot depicting the bacterial load in H37Rv-mCherry–infected peritoneal macrophages from NCoR1^fl/fl^ and NCoR1^MyeKO^ mice (*n* = 6 mice). (N, O) Western blot image showing the NCoR1 protein level kinetics with quantification (bar plot) in control and NCoR1 KD cDC1 (conventional type I dendritic cells) upon H37Rv infection. For normalisation, β-actin was used as housekeeping control (*n* = 3). (P) Scatter plot showing the H37Rv bacterial load in control and NCoR1 KD cDC1 at 24 h time point as estimated by CFU assay (*n* = 9). (Q) Histogram from FACS analysis showing the MFI shifts of H37Rv-mCherry–infected control and NCoR1 KD cDC1 at 24 h post infection (*n* = 3). (R) Bar plot demonstrating the quantification of MFI shifts from 3 independent biological replicates of H37Rv-mCherry–infected control and NCoR1 KD cDC1 at 24 h post infection (*n* = 3). *p < 0.05, *p < 0.01, and ***p < 0.001 using paired and unpaired two-tailed Student’s *t* test. Where n represents independent biological replicates. The data underlying this figure are available in S4 Table and S1 Data. Western blot raw images can be found in S1 Raw Image. BMDM, bone marrow-derived macrophage; KD, knockdown; MFI, mean fluorescence intensity.

To decipher the role of NCoR1 in *Mtb* infection, we developed a stable *NCoR1* gene knockdown (KD) THP1 mo-MΦ line using lentivirus-mediated shRNA transduction. To assess the efficiency of shRNA-mediated NCoR1 depletion, we performed kinetics of NCoR1 expression in H37Rv-infected control and NCoR1 KD mo-MΦ. We found a significant depletion of NCoR1 at transcript as well as protein levels at all time points (**Figs 2A, 2B, and S2A**). To assess the impact of NCoR1 depletion on *Mtb* load, we infected the control and NCoR1 KD mo-MΦ by pathogenic strain of *Mycobacterium tuberculosis*, i.e., H37Rv and nonpathogenic laboratory strain *M*. *smegmatis* to quantify the bacterial load using CFU assay at 24 h post infection. We found that NCoR1 KD mo-MΦ harbours more H37Rv or *M*. *smegmatis* burden as compared to control cells (**Figs 2C and S2B**). In addition, we confirmed these results by flow cytometry using mCherry-tagged H37Rv or *M*. *smegmatis*, where we observed similar and significantly increased percent infection along with their respective mean fluorescence intensity (MFI) shifts for H37Rv or *M*. *smegmatis* in NCoR1 KD mo-MΦ (**Figs 2D, 2E, and S2C–S2F**). Further, to overcome the off-target effects, NCoR1 transcript levels were also checked with a different shRNA (**S2G Fig**). Similar results were confirmed using shRNA3-mediated NCoR1 KD mo-MΦ as well (**S2H, S2K and S2L Figs**). We also quantified phagocytosis rate kinetics to observe infection difference at early time points between control and NCoR1 KD cells using yellow-green latex beads and *M*. *smegmatis* infection at 10 min, 30 min, and 60 min time points using flow cytometry and CFU assay [43]. We did not observe any phagocytosis difference at any of the early time points, suggesting the role of NCoR1 in controlling *Mtb* survival independent of phagocytosis rate (**Figs 2F–2H, S2I and S2J**). Next, we asked whether similar phenomena were preserved in primary cells as well. We generated myeloid specific (macrophages and DCs) conditional NCoR1 knockout (NCoR1^MyeKO^) mice by breeding homozygous NCoR1^fl/fl^-CD11c-Cre C57BL/6 mice with LysM-Cre FvB mice. After more than 10 generations of crosses, the genotyped homozygous double (macrophage and DC) NCoR1 KO mice and matched control littermates were used for experiments (**S2M Fig**) [40]. To exclude bias and confirm deletion of NCoR1 in myeloid cells with respect to other cell types, we sorted CD11c^+^ and CD11c^-^ populations and found *Ncor1* transcript levels to be ablated in the CD11c^+^ fraction (**S2N Fig**) [40,44]. We analysed the expression of NCoR1 in BMDMs from NCoR1^MyeKO^ mice, where we found significant depletion of NCoR1 at both the protein and transcript levels (**Fig 2I–2K**). Next, we investigated the levels of *Mtb* infection in BMDMs and peritoneal macrophages and found similar increased susceptibility in our CFU assays for NCoR1 KO mice (**Fig 2L and 2M**). To further substantiate our observations in DCs, we infected previously characterised stable control and NCoR1 KD cDC1 line [40]. NCoR1 depletion at the protein level was confirmed upon H37Rv infection (**Fig 2N and 2O**). These cells also depicted similar increased susceptibility to H37Rv and *M*. *smegmatis* infection in CFU assays (**Figs 2P, S2O and S2P**). The MFI also depicted a significantly increased shift for H37Rv infection in NCoR1 KD cDC1 as compared to controls (**Fig 2Q and 2R**). Overall, our results clearly demonstrated that NCoR1 depletion plays an essential role in myeloid cells (macrophages as well as DCs) to augment *Mtb* infection.

### NCoR1^MyeKO^ mice depicted enhanced *Mycobacterium* infection in vivo

**Fig 3:**
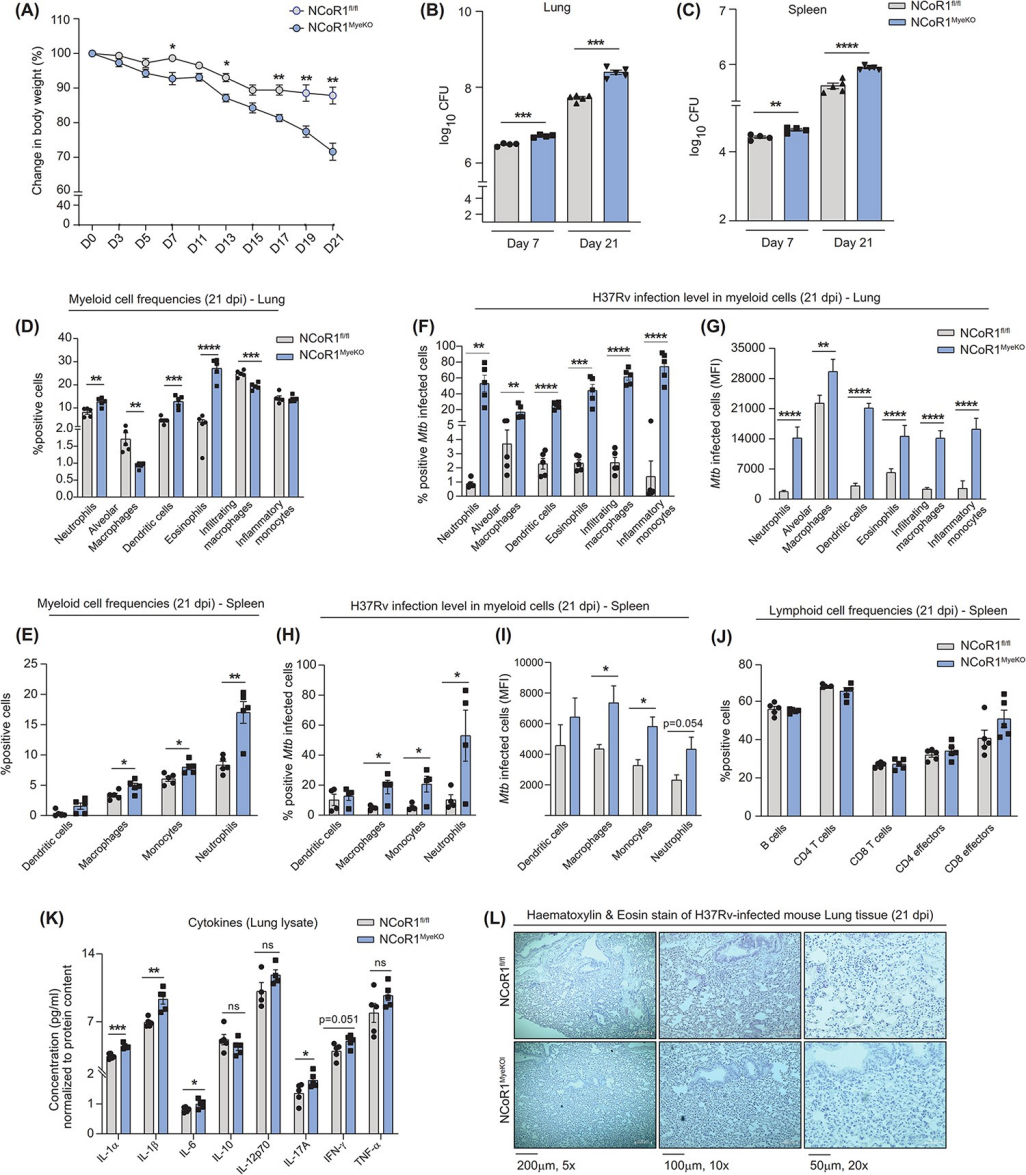
Myeloid specific NCoR1 deletion exacerbates *Mycobacterium* infection in mice. (A) Line graph showing the percent reduction in body weight upon H37Rv infection in NCoR1^fl/fl^ and NCoR1^MyeKO^ mice at regular intervals till day 21 post infection (*n* = 5 mice). (B, C) Bar plots showing the bacterial load in the lung tissues of H37Rv-infected NCoR1^fl/fl^ and NCoR1^MyeKO^ mice at day 7 and day 21 post infection by CFU assay in lung and spleen. Data is presented as the median log_10_CFU (*n* = 4–5). (D) Bar plots showing the percent positive myeloid cell subtypes gated on CD45 positive cells isolated from lung tissues of NCoR1^fl/fl^ and NCoR1^MyeKO^ mice on day 21 post infection. Strategy used to gate H37Rv-infected macrophages in FACS is shown in S3C Fig (*n* = 5). (E) Bar plots showing the percent positive myeloid cell subtypes gated on CD45 positive cells isolated from spleen tissues of NCoR1^fl/fl^ and NCoR1^MyeKO^ mice on day 21 post infection. Strategy used to gate H37Rv-infected macrophages in FACS is shown in S3D Fig (*n* = 5). (F) Bar plots showing the percentage of GFP-tagged H37Rv infection in neutrophils, alveolar macrophages, dendritic cells, eosinophils, infiltrating macrophages, and inflammatory monocytes, and (G) corresponding MFI shifts in the cells isolated from lung tissues of NCoR1^fl/fl^ and NCoR1^MyeKO^ mice on day 21 post infection (*n* = 5). (H) Bar plot showing the percentage of GFP-tagged H37Rv infection in dendritic cells, macrophages, monocytes, and neutrophils, and (I) corresponding MFI shifts in the cells isolated from spleen tissues of NCoR1^fl/fl^ and NCoR1^MyeKO^ mice on day 21 post infection (*n* = 4). (J) Bar plot showing the percent positive B cell and T cell subtypes gated on CD45 positive cells isolated from splenic tissues of NCoR1^fl/fl^ and NCoR1^MyeKO^ mice on day 21 post infection. Strategy used to gate H37Rv-infected macrophages in FACS is shown in S3E Fig (*n* = 5). (K) Bar plot showing the level of different inflammatory cytokines in the lung tissue lysate of NCoR1^fl/fl^ and NCoR1^MyeKO^ mice on day 21, normalised to protein content (*n* = 5). (L) Representative HE staining image showing infiltration of cells in the lung tissue of NCoR1^fl/fl^ and NCoR1^MyeKO^ mice on day 21. *p < 0.05, *p < 0.01, and ***p < 0.001 using an unpaired, two-tailed Student’s *t* test. Where n represents the total number of used mice. The data underlying this figure are available in S1 Data. HE, haematoxylin and eosin; MFI, mean fluorescence intensity.

To establish the physiological impact of NCoR1 in *Mtb* pathogenicity and virulence in vivo, we infected control NCoR1^fl/fl^ and NCoR1^MyeKO^ mice with 10^5^ H37Rv (GFP-tagged) (**S3A Fig**) [45]. The body weights of infected mice were measured at regular intervals, and we found that NCoR1^MyeKO^ animals started to show a significant reduction in body weight from day 7 post infection as compared to floxed control mice, which was further reduced (day 17 to day 21) in subsequent days (**Fig 3A**). In addition, we observed profoundly enhanced splenomegaly in NCoR1^MyeKO^ animals at day 21 post infection (**S3B Fig**). Next, we estimated the bacterial load in the lungs and spleen of infected mice. NCoR1^MyeKO^ mice showed increased bacterial load at day 7 which was further increased at day 21 post infection (**Fig 3B and 3C**). We also examined the myeloid cell frequencies in lung and spleen using specific markers and gating strategies (**S3C and S3E Fig**) [46]. We found increased frequencies and cell numbers of neutrophils, dendritic cells, and eosinophils with decreased levels of alveolar and infiltrating macrophages at day 21 post infection per lung of NCoR1^MyeKO^ as compared to control (**Figs 3D and S3D**). Whereas, in spleen, we did not observe a significant difference in DCs but a significantly increased frequency of macrophages, monocytes, and neutrophils were observed in NCoR1^MyeKO^ (**Fig 3E**). The cell numbers of the above myeloid cell types also showed similar trends in the spleen (**S3F Fig**). Next, we evaluated the *Mtb* infection in the lung myeloid cells, where we observed elevated *Mtb* infection in neutrophils, alveolar macrophages, dendritic cells, eosinophils, infiltrating macrophages, and inflammatory monocytes in the lung of NCoR1^MyeKO^ as compared to NCoR1^fl/fl^ mice (**Figs 3F, 3G, and S3D**). Further, similar increased frequency and cell numbers of *Mtb-*infected dendritic cells, macrophages, monocytes, and neutrophils were obtained in the spleen of NCoR1^MyeKO^ mice (**Figs 3H, 3I, and S3F**). In addition, we also evaluated the lymphocyte compartments, where we did not observe any difference in the frequencies and cell numbers of B and T cell subtypes (**Figs 3J, S3G, and S3H**). Further, we asked whether NCoR1^MyeKO^ mice have increased inflammation culminating into higher susceptibility for *Mtb* infection. For which we performed a bio-plex assay to quantify various cytokines in the lung lysates and found NCoR1^MyeKO^ mice to have significantly high levels of inflammatory cytokines like IL-1α, IL-1β, IL-6, and IL-17A (**Fig 3K**). In addition, we examined the infiltration of cells in the lung tissue using haematoxylin and eosin (HE) staining. We found increased infiltration of cells in the *Mtb*-infected lung section of NCoR1^MyeKO^ mice as compared to control (**Fig 3L**) [47]. These results were consistent with other previous reports showing that disrupted autophagy can elevate inflammatory mediators [48,49]. These results demonstrated that NCoR1 is a crucial host factor in controlling TB disease, and its ablation exacerbates *Mtb* pathogenesis.

### NCoR1 regulates autophagy induction during *Mtb* infection

**Fig 4:**
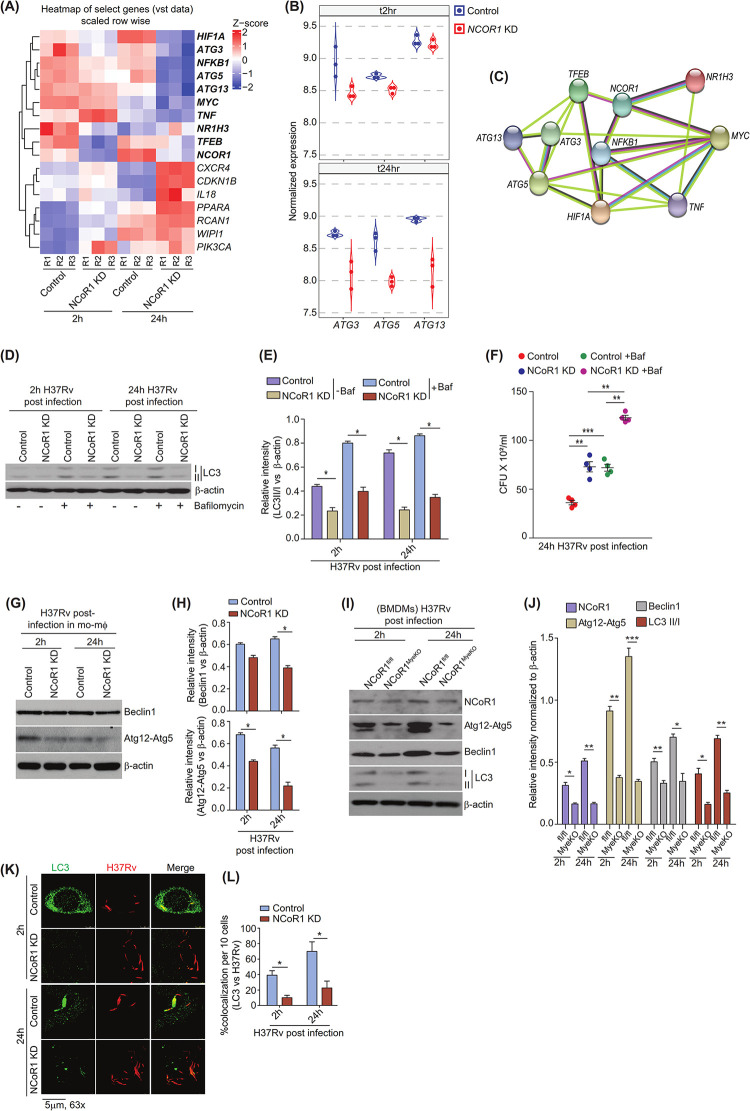
NCoR1 regulates autophagy induction during *Mtb* infection. (A) Heat map showing the top differential expressed genes related to autophagy function in the RNA-seq data of control and NCoR1 KD human monocytic THP-1 differentiated macrophages at 2 h and 24 h post infection (*n* = 3). (B) Violin plot depicting the normalised transcript expression of ATGs in control and NCoR1 KD human monocytic THP-1 differentiated macrophages at 2 h and 24 h post infection (*n* = 3). (C) String network analysis showing the association of NCoR1 with top DEGs found in NCoR1 KD human monocytic THP-1 differentiated macrophages vs. control cells at 2 h and 24 h post infection (*n* = 3). (D, E) Representative western blot image depicting the LC3-II:LC3-I level in H37Rv-infected control and NCoR1 KD human monocytic THP-1 differentiated macrophages at 2 h and 24 h post infection, before and after treatment with bafilomycin. Corresponding densitometric analysis (bar plots) depicting the quantitation and statistics from the western blot images of 3 independent biological replicates. For normalisation, β-actin was used as housekeeping control. LC3-II density versus LC3-I was quantified followed by normalisation with β-actin (*n* = 3). (F) Scatter plot showing the H37Rv bacterial load in control and NCoR1 KD human monocytic THP-1 differentiated macrophages by CFU assay at 24 h post infection, before and after treatment with bafilomycin (*n* = 4). (G, H) Western blot representative image depicting the protein levels of Beclin1 and ATG12-5 in H37Rv-infected control and NCoR1 KD human monocytic THP-1 differentiated macrophages at 2 h and 24 h post infection. Corresponding densitometric analysis (bar plots) showing the quantitation and statistics from the western blot images of 3 independent biological replicates. For normalisation, β-actin was used as housekeeping control (*n* = 3). (I) Western blot representative picture showing the levels of NCoR1, Atg12-Atg5, Beclin1, and LC3-II:LC3-I in H37Rv-infected BMDMs generated from NCoR1^fl/fl^ and NCoR1^MyeKO^ mice at 2 h and 24 h post infection. For normalisation, β-actin was used as housekeeping control. LC3-II density versus LC3-I was quantified followed by normalisation with β-actin (*n* = 4 mice). (J) Bar plot showing the densitometric quantification from western blot images for NCoR1, ATG12-ATG5, BECLIN1, and LC3 in H37Rv-infected BMDMs generated from NCoR1^MyeKO^ and NCoR1^fl/fl^ mice at 2 h and 24 h post infection. For normalisation, β-actin was used as housekeeping control (*n* = 4 mice). (K) Confocal microscopy showing the colocalization of H37Rv with LC3 protein in control and NCoR1 KD human monocytic THP-1 differentiated macrophages at 2 h and 24 h post infection (*n* = 3). (L) Bar plot depicting the quantification of confocal images from 3 independent biological replicates for the colocalization of H37Rv with LC3 in control and NCoR1 KD human monocytic THP-1 differentiated macrophages. Ten cells from each biological replicate were analysed for calculating the colocalization percentage (*n* = 3). *p < 0.05, *p < 0.01, and ***p < 0.001 using paired and unpaired two-tailed Student’s *t* test. Where n represents independent biological replicates. The data underlying this figure are available in S1 Table and S1 Data. Western blot raw images can be found in S1 Raw Image. BMDM, bone marrow-derived macrophage; DEG, differentially expressed gene; KD, knockdown.

Next, we intended to look into the mechanism of dysregulated host responses upon NCoR1 depletion leading to increased *Mycobacterium* pathogenesis. To gain a global insight into the mechanistic aspect, we performed RNA-seq analysis of *Mtb*-infected control and NCoR1 KD mo-MΦ at 2 h and 24 h post infection (**S1 Table**). We found a set of genes involved in the autophagy process that were significantly down, namely, ATG3, ATG5, and ATG13. Hypoxic molecule HIF-1α (stabilisation has antimicrobial activity) was also found to be decreased in the NCoR1 KD condition at 24 h post infection (**Fig 4A and 4B and S2 Table**). In addition, inflammatory mediators like TNFα and NFκB also showed diminished expression (**Fig 4A**). It has already been documented that autophagy plays a central role in host defence mechanisms to clear the intracellular pathogens including *Mtb* by augmenting intracellular cargos to destroy in phagolysosomes [50,51]. Moreover, NCoR1 showed a strong association with the autophagy pathway in string network analysis as well (**Fig 4C**). Mo-MΦ-mediated autophagy induces LC3 lipidation (LC3II) at an early time point upon H37Rv infection, which is an important event to control bacterial infection through an optimal level of autophagy flux [52]. Next, to confirm the same, we treated H37Rv-infected control and NCoR1 KD mo-MΦ with and without bafilomycin. We found that the LC3-II: LC3-I ratio was significantly reduced in NCoR1 KD mo-MΦ as compared to control cells confirming the observation from RNA-seq data (**Fig 4D and 4E**). We also found that LC3-II:LC3-I ratio was significantly elevated in the bafilomycin treated control mo-MΦ cells as compared to untreated cells, suggesting that autophagy flux is intact (**Fig 4D and 4E**). On the other hand, in the NCoR1 KD mo-MΦ, the LC3-II:LC3-I was reduced in untreated NCoR1 KD mo-MΦ compared to control cells, which showed slight but insignificant increase upon bafilomycin treatment (**Fig 4D and 4E**). In addition, bafilomycin treatment aggravated the NCoR1 mediated support of intracellular *Mtb* growth in our CFU and flow cytometry analysis, suggesting a role of NCoR1 in autophagy induction process (**Figs 4F and S4A–S4C**). This suggested that the overall autophagy process is abrogated in NCoR1 depleted cells. To substantiate these observations, we analysed the ATG12-ATG5 conjugate and Beclin1 proteins that are shown to be crucial for auto-phagosome formation and nucleation in active autophagy in various reports [53,54]. We found that NCoR1 KD mo-MΦ showed significantly decreased Beclin1 and ATG12-ATG5 protein levels upon H37Rv infection (**Fig 4G and 4H**). Moreover, we found similar decreased levels of autophagy proteins in BMDMs generated from NCoR1^MyeKO^ mice and in cDC1 (**Figs 4I, 4J, S4D and S4E**). We also asked whether NCoR1 KD mo-MΦ cells have reduced autophagy already at basal levels or is it H37Rv specific. We compared LC3 expression in H37Rv post-infected cells at 2 h to that in uninfected control, where we observed reduced LC3-II:LC3-I ratios only after infection (**S4F Fig**) [52]. Next, we wanted to investigate the entrapment of H37Rv with LC3. We observed a strong colocalization of LC3 with H37Rv in control mo-MΦ cells, whereas in NCoR1 KD mo-MΦ and BMDMs from NCoR1^MyeKO^ mice showed diminished colocalization of these proteins with a concomitant increase in bacterial load (**Figs 4K, 4L, S4G and S4H**). These findings suggested the dependency of myeloid cells on NCoR1 for the induction of an optimum autophagy flux upon *Mtb* infection. However, we cannot ignore the fact that disrupted lysosome mediated *Mtb* survival could also contribute in addition to inhibited autophagy in NCoR1 depleted myeloid cells as both pathways are known to be regulated by TFEB.

### NCoR1 regulates mTOR-TFEB axis to control autophagy induction and lysosomal biogenesis in myeloid cells

**Fig 5:**
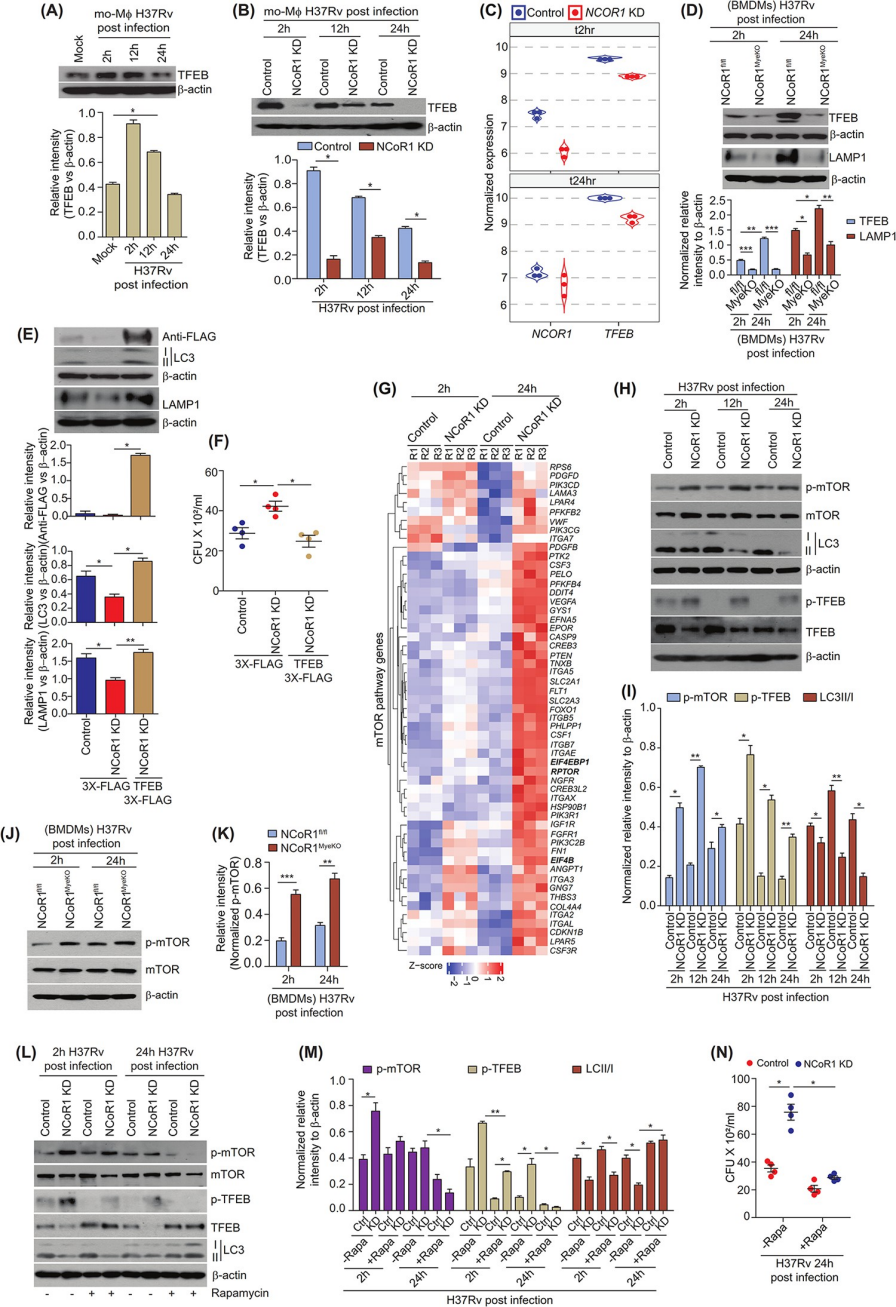
NCoR1 regulates mTOR-TFEB axis to control autophagy induction and lysosomal biogenesis in myeloid cells. (A) Representative western blot image along with bar plots for densitometric analysis depicting the TFEB protein kinetics (2 h, 12 h, and 24 h) in the H37Rv-infected human monocytic THP-1 differentiated macrophages. For normalisation, β-actin was used as housekeeping control (*n* = 3). (B) Western blot image depicting the levels of TFEB protein in H37Rv-infected control and NCoR1 KD human monocytic THP-1 differentiated macrophages at 2 h, 12 h, and 24 h post infection. Corresponding bar plots showing the densitometric analysis from 3 independent biological replicates. For normalisation, β-actin was used as housekeeping control (*n* = 3). (C) Violin plot depicting the normalised transcript expression of TFEB in RNA-seq data of control and NCoR1 KD human monocytic THP-1 differentiated macrophages at 2 h and 24 h post infection (*n* = 3). (D) Representative western blot image with densitometric analysis showing the TFEB and LAMP1 levels in the BMDMs from NCoR1^fl/fl^ and NCoR1^MyeKO^ mice at 2 h and 24 h post H37Rv infection. For normalisation, β-actin was used as housekeeping control (*n* = 4 mice). (E) Western blot image along with densitometric analysis showing the levels of LC3, LAMP1, and TFEB-flag in H37Rv-infected NCoR1 KD human monocytic THP-1 differentiated macrophages with or without overexpression of exogenous flag-tagged TFEB at 24 h post infection. For normalisation, β-actin was used as housekeeping control (*n* = 3). (F) Scatter plot demonstrating the H37Rv bacterial load by CFU assay in H37Rv-infected NCoR1 KD human monocytic THP-1 differentiated macrophages with or without exogenous overexpression of flag-tagged TFEB at 24 h post infection (*n* = 4). (G) Heat map showing the DEGs related to mTOR pathway in RNA-seq data of control and NCoR1 KD human monocytic THP-1 differentiated macrophages at 2 h and 24 h post infection (*n* = 3). (H) Representative western blot image depicting the kinetics (2 h, 12 h, 24 h) of phospho-mTOR (p-mTOR), mTOR, phospho-TFEB (p-TFEB), TFEB, and LC3-II:LC3-I protein levels in H37Rv-infected control and NCoR1 depleted human monocytic THP-1 differentiated macrophages (*n* = 3). (I) Bar plot showing the densitometric quantification of p-mTOR, mTOR, p-TFEB, TFEB, and LC3 protein bands from 3 independent biological replicates in H37Rv-infected human control and NCoR1 KD monocytic THP-1 differentiated macrophages at 2 h, 12 h, and 24 h post infection. p-mTOR and p-TFEB were normalised first with total protein levels and then with housekeeping control β-actin. LC3-II density versus LC3-I was quantified followed by normalisation with β-actin (*n* = 3). (J) Representative western blot image depicting the protein levels of p-mTOR and total mTOR in H37Rv-infected BMDMs generated from NCoR1^fl/fl^ and NCoR1^MyeKO^ mice at 2 h and 24 h post infection. For normalisation, β-actin was used as housekeeping control (*n* = 4 mice). (K) Bar plot showing the densitometric quantification of p-mTOR levels from 3 independent biological replicates in H37Rv-infected BMDMs generated from NCoR1^fl/fl^ and NCoR1^MyeKO^ mice at 2 h and 24 h post infection. p-mTOR was normalised first with total m-TOR followed by normalisation with β-actin (*n* = 4 mice). (L) Western blot representative image depicting the p-mTOR, mTOR, p-TFEB, TFEB, and LC3-II:LC3-I levels in H37Rv-infected control and NCoR1 KD human monocytic THP-1 differentiated macrophages at 2 h and 24 h post infection, with and without rapamycin treatment (*n* = 3). (M) Bar plot depicting the densitometric quantification of normalised p-mTOR, p-TFEB, and LC3 protein bands from 3 independent biological replicates. The p-mTOR and p-TFEB levels were normalised first with their respective total protein levels and then with housekeeping control β-actin. LC3-II versus LC3-I levels were quantified followed by normalisation with β-actin (*n* = 3). (N) Scatter plot showing the bacterial load in H37Rv-infected control and NCoR1 KD human monocytic THP-1 differentiated macrophages by CFU assay at 24 h post infection, with and without treatment of rapamycin (*n* = 4). *p < 0.05, *p < 0.01, and ***p < 0.001 using paired and unpaired two-tailed Student’s *t* test. Where n represents independent biological replicates. The data underlying this figure are available in S1 Table and S1 Data. Western blot raw images can be found in S1 Raw Image. BMDM, bone marrow-derived macrophage; DEG, differentially expressed gene; KD, knockdown; TFEB, transcription factor EB.

The TFEB is a known master regulator of autophagy induction and maturation controlling genes [12,55]. We argued whether disrupted autophagy machinery in NCoR1 depleted myeloid cells is TFEB dependent or independent. Thus, we sought the expression kinetics of TFEB protein in H37Rv-infected wild-type mo-MΦ and cDC1, where we observed similar expression kinetics of TFEB as NCoR1, suggesting a possible association of NCoR1 and TFEB (**Figs 5A, S5A and S5B**). Moreover, we observed an increased expression of NCoR1 and TFEB in CD11c^+^ and F4/80^+^ cells of *Mtb*-infected lung tissue of mice (**S5C and S5D Fig**). Next, we investigated the levels of TFEB protein in H37Rv-infected control and NCoR1 depleted mo-MΦ and cDC1. We found that TFEB expression was reduced at an early point of infection in NCoR1-deficient conditions, indicating a crucial role of NCoR1 in TFEB functionality (**Figs 5B, 5C, S5E, and S5F**). We also observed similar reduced levels of TFEB with increased infection in NCoR1^MyeKO^ BMDMs as compared to control (**Figs 5D, S5G and S5H**). It is well reported that TFEB directly controls the expression of genes involved in autophagy and lysosomal pathways [20,56,57]. We also found reduced LAMP1 expression and its colocalization with *Mtb* in NCoR1^MyeKO^ BMDMs as compared to control NCoR1^fl/fl^ BMDMs (**Figs 5D, S5I and S5J**). To ascertain the role of TFEB in compromised autophagy and lysosomal biogenesis, we overexpressed the flag-tagged TFEB in NCoR1 KD mo-MΦ followed by H37Rv infection for 24 h. We analysed the expression of LC3-II protein and LAMP1 in TFEB overexpressed NCoR1 KD mo-MΦ. We found restored levels of LC3-II and LAMP1, indicating the dependency of NCoR1 on TFEB in regulating autophagy and lysosomal biogenesis upon *Mtb* infection (**Fig 5E**). In addition, overexpression of TFEB in NCoR1 KD mo-MΦ significantly rescued the bacterial clearance (**Fig 5F**). Next, we asked whether host cells regulate the NCoR1-mediated autophagy process regardless of active *Mtb* infection or any other stress. Control and NCoR1 KD mo-MΦ were starved and infected with heat-killed H37Rv, they showed similar increased levels of NCoR1 along with TFEB and LC3-II:LC3-I in control mo-MΦ, whereas their expression was significantly reduced in starvation and heat-killed *Mtb* treated NCoR1 KD mo-MΦ indicating a possible role of NCoR1 in the autophagy induction process (**S5K–S5N Fig**). Induction of NCoR1 was weaker in starved conditions as compared to *Mtb* infection in mo-MΦ. Next, we investigated the mechanisms underlying the regulation of TFEB through NCoR1. It has been shown in various reports that mTOR regulates the TFEB activity by phosphorylation [21,58,59]. Our RNA-seq data depicted similar up-regulation of mTOR pathway genes in NCoR1 KD mo-MΦ at 24 h post *Mtb* infection (**Fig 5G and S1 and S2 Tables**). To confirm our observation, we analysed the activated mTOR, i.e., phospho-mTOR upon H37Rv infection. We found significantly increased phosphorylation of mTOR in H37Rv-infected NCoR1 KD mo-MΦ as compared to control cells (**Fig 5H and 5I**). At the same time, we observed increased phospho-TFEB, an inactive form of TFEB, in the H37Rv-infected NCoR1 KD mo-MΦ along with decreased LC3 level, consistent with previous reports (**Fig 5H and 5I**) [21]. These data indicate possible direct control of elevated mTOR signalling on observed reduced TFEB activity in NCoR1 KD myeloid cells. Moreover, we found similar increased phospho-mTOR in BMDMs from NCoR1^MyeKO^ mice (**Fig 5J and 5K**). Next, we hypothesised that mTOR inhibition could rescue the active TFEB and restore autophagy in NCoR1 KD mo-MΦ, leading to decreased bacterial load. We treated the cells with rapamycin, which is well known to inhibit the mTOR activity [60]. Indeed, rapamycin treated with NCoR1 KD mo-MΦ showed reduced phospho-mTOR, confirming the inhibitory effect of rapamycin on mTOR activity (**Fig 5L and 5M**). We found decreased phospho-TFEB and increased TFEB with LC3-II levels in mTOR inhibited condition of NCoR1 KD mo-MΦ at 2 h and 24 h post H37Rv infection (**Fig 5L and 5M**). Moreover, we observed a comparable elevation of TFEB level when we used Torin1, which is a more specific mTOR inhibitor. At the same time, we observed a down-regulation of phospho-TFEB and phospho-mTOR (**S5O and S5P Fig**) [58]. In addition, rapamycin treated NCoR1 KD mo-MΦ showed decreased H37Rv bacterial load compared to untreated conditions in CFU and flow cytometry analysis (**Figs 5N, S5Q and S5R**). These findings elucidated how NCoR1 impacts the mTOR-TFEB axis, which in turn regulates the genes involved in autophagy and the lysosomal pathway in myeloid cells.

### NCoR1 regulates mTOR activity by fine-tuning cellular ATP-AMPK levels

**Fig 6:**
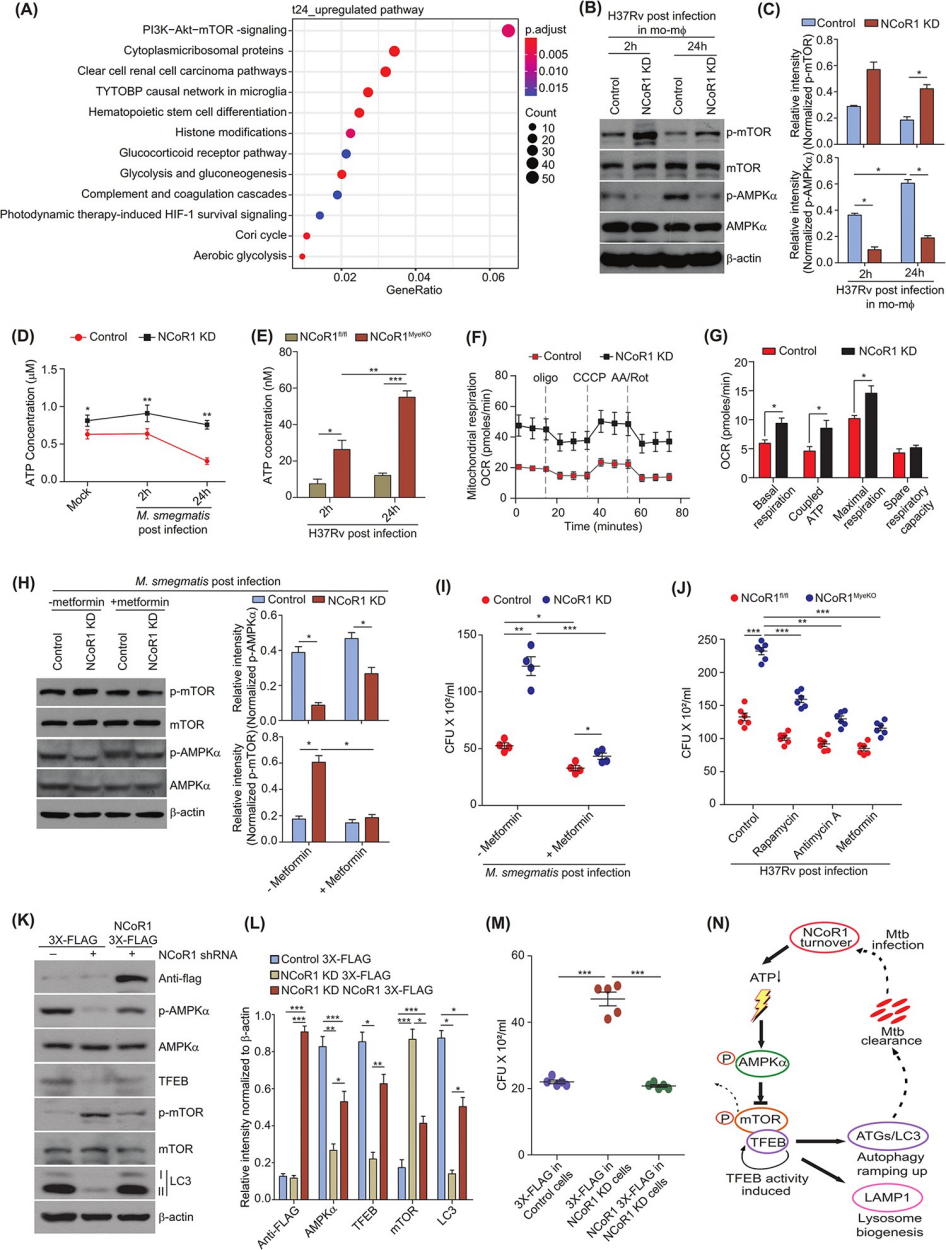
NCoR1 regulates mTOR activity by fine-tuning cellular ATP-AMPK levels. (A) Pathway enrichment analysis showing the top pathways enriched for the list of genes significantly up-regulated in RNA-seq data of NCoR1 KD human monocytic THP-1 differentiated macrophages at 24 h post infection with H37Rv (*n* = 3). (B, C) Representative western blot image depicting the p-mTOR, p-AMPKα along with total mTOR, AMPKα in H37Rv-infected control and NCoR1 KD human monocytic THP-1 differentiated macrophages at 2 h and 24 h post infection. Corresponding bar plots showing the densitometric analysis from western blots of 3 independent biological replicates (*n* = 3). (D) Line graph showing the intracellular ATP levels in *M*. *smegmatis*-infected control and NCoR1 KD human monocytic THP-1 differentiated macrophages at 2 h and 24 h post infection (*n* = 3). (E) Bar graph depicting the intracellular ATP levels in NCoR1^fl/fl^ and NCoR1^MyeKO^ BMDMs at 2 h and 24 h post H37Rv infection (*n* = 4 mice). (F) Representative seahorse assay line graph showing the OCR levels upon sequential injections with Oligomycin, CCCP, and Rotenone/Antimycin A, measured in *M*. *smegmatis*-infected control and NCoR1 KD human monocytic THP-1 differentiated macrophages at 2 h and 24 h post infection (*n* = 4). (G) Bar plot depicting the quantification of OCR levels as basal respiration, coupled ATP, maximal respiration, and spare respiratory capacity, measured by seahorse assay, of *M*. *smegmatis-*infected control and NCoR1 KD human monocytic THP-1 differentiated macrophages at 2 h and 24 h post infection (*n* = 4). (H) Western blot representative image with corresponding densitometric analysis depicting the p-mTOR, p-AMPKα along with total mTOR, AMPKα protein level in *M*. *smegmatis*-infected control and NCoR1 KD human monocytic THP-1 differentiated macrophages at 24 h post infection, with and without metformin treatment. All phosphorylated proteins were first normalised with its total form followed by housekeeping control β-actin (*n* = 3). (I) Scatter plot showing the *M*. *smegmatis* bacterial load in control and NCoR1 KD human monocytic THP-1 differentiated macrophages by CFU assay at 24 h post infection, with and without metformin treatment (*n* = 4). (J) Scatter plot showing the bacterial load in H37Rv-infected BMDMs at 24 h post infection by CFU assay, with and without treatment of antimycin A, rapamycin, and metformin. BMDMs were generated from NCoR1^MyeKO^ and NCoR1^fl/fl^ mice (*n* = 6 mice). (K) Western blot image demonstrating the protein levels of exogenously expressed NCoR1-flag and its impact on p-AMPKα, AMPKα, TFEB, p-mTOR, total mTOR, and LC3-II:LC3-I in H37Rv-infected NCoR1 KD human monocytic THP-1 differentiated macrophages (*n* = 3). (L) Bar plot showing the densitometric quantitation of western blot bands of NCoR1-flag, p-AMPKα, TFEB, p-mTOR, and LC3 protein levels from 3 independent biological replicates of H37Rv-infected NCoR1 KD human monocytic THP-1 differentiated macrophages complemented with exogenous NCoR1-flag. All phosphorylated proteins were first normalised with its total form followed by housekeeping control β-actin (*n* = 3). (M) Scatter plot showing the bacterial load in H37Rv-infected control, NCoR1 KD, and exogenous NCoR1-flag overexpressed NCoR1 KD in human monocytic THP-1 differentiated macrophages by CFU assay at 24 h post infection (*n* = 5). (N) Cartoon diagram showing the proposed mechanism of NCoR1 regulating *Mtb* pathogenesis in myeloid cells. *p < 0.05, *p < 0.01, and ***p < 0.001 using paired and unpaired two-tailed Student’s *t* test. Where n represents independent biological replicates. The data underlying this figure are available in S2 Table and S1 Data. Western blot raw images can be found in S1 Raw Image. AMPK, AMP-activated protein kinase; ATP, adenosine triphosphate; BMDM, bone marrow-derived macrophage; KD, knockdown.

mTOR has been shown to sense different kinases to integrate different signalling axes to tailor key physiological processes based on specific local cues [61,62]. Using differentially expressed genes (DEGs) from RNA-seq data (**S1 Table**), we performed pathway enrichment analysis to comprehend the molecular signalling under the NCoR1 depleted condition. We found that the PI3K-AKT-mTOR pathway was the top signalling pathway up-regulated at 24 h in NCoR1 KD cells, while the proteasome pathways were down-regulated. (**Figs 6A and S6A and S2 Table**). It has been well reported that mTORC regulates autophagy, which is known to be negatively regulated by phosphorylated AMPK [63]. Next, we investigated the levels of phospho-AMPK (p-AMPK) in NCoR1 KD mo-MΦ that showed significantly increased phospho-mTOR. NCoR1 KD mo-MΦ showed lower AMPK activity at 2 h and 24 h post *Mycobacterium* infection, which is consistent with the mTOR activity (**Fig 6B and 6C**). AMPK is a key sensor for dynamic ATP concentration in cells and responds inversely to cellular energy state with respect to mTOR [15,64]. Moreover, NCoR1 is well reported to modulate the expression of different metabolic genes [26,65,66]. Recently, it has been reported that liver-specific NCoR1 knock out (KO) mice showed elevated level of ATP and increased mitochondrial reactive oxygen species, which subsequently blocks the process of diethylnitrosamine (DEN)-induced HCG in mice [29]. We therefore considered whether NCoR1 KD mo-MΦ has a similar kind of perturbation at cellular energy levels upon bacterial infection or not. We found that NCoR1 KD mo-MΦ has elevated intracellular levels of ATP at 2 h and 24 h of *M*. *smegmatis* infection as compared to control cells (**Fig 6D**). In addition, BMDMs from NCoR1^MyeKO^ also showed increased ATP level as compared to WT BMDMs (**Fig 6E**). Next, we addressed the metabolic state in NCoR1 depleted cells using the Seahorse extracellular flux assay, where we found a similarly increased coupled ATP production rate through elevated OXPHOS in NCoR1 KD mo-MΦ (**Figs 6F, 6G, and S6B**). Similar observations are also attained in NCoR1 ablated cDC1 DCs reported previously [30]. To identify the role of AMPK/mTOR signalling culminating into dysregulated autophagy in NCoR1 KD mo-MΦ upon mycobacterial infection, we treated the cells with metformin, an AMPK activator. We found that metformin-treated NCoR1 KD mo-MΦ showed increased AMPK activity which in turn decreased the levels of p-mTOR leading to protection from *M*. *smegmatis* infection suggesting the NCoR1 mediated regulation of AMPK/mTOR pathway controlling the mycobacterial load through autophagy (**Fig 6H and 6I**). We further sought to confirm the role of NCoR1 mediated increased ATP levels in the compromised autophagy process leading to increased *Mtb* infection. We treated *M*. *smegmatis* infected cells with antimycin-A, an inhibitor of complex III of ETC, to deplete the ATP level at 6 h post infection. Antimycin-A treatment reduced the ATP levels and a subsequent reduction in infection levels were observed in NCoR1 KD mo-MΦ upon *M*. *smegmatis* infection (**S6C–S6G Fig**). Moreover, we observed that antimycin-A treated NCoR1 KD mo-MΦ had recovered AMPK activity, which consequently led to decreased levels of p-mTOR leading to increased TFEB and LC3-II:LC3-I (**S6H and S6I Fig**). In all the experiments, more than 90% cell viability upon antimycin-A treatment (0.125 μm to 0.25 μm) was confirmed by FACS (**S6J Fig**). We further confirmed our rescue experiments in BMDMs generated from NCoR1^MyeKO^ and found significant rescue from *Mtb* infection upon rapamycin, antimycin-A, and metformin treatments (**Fig 6J**). To complement our results that NCoR1 is a key candidate to control the pathogenesis of *Mtb* in the host and it could be a putative target in controlling *Mtb* infection, we overexpressed exogenous NCoR1 in NCoR1 depleted mo-MΦ. We found that exogenous delivery of NCoR1 was sufficient to restore the compromised AMPK/mTOR signalling leading to recovered LC3-II levels (**Fig 6K and 6L**). Moreover, exogenous NCoR1 complementation rescued the NCoR1 KD mo-MΦ from the susceptibility to *Mtb* infection (**Fig 6M**). Altogether, these results confirmed and propose an important role of NCoR1 in balancing the cellular ATP sensor AMPK-mTOR-TFEB pathway upon *Mtb* infection for the optimal induction of TFEB activity, hence, its clearance through auto-phagolysosome pathway (**Fig 6N**).

## Discussion

This investigation is the first report depicting the role of NCoR1 mediated fine-tuning of the auto-phagolysosomal pathway in regulating *Mtb* pathogenesis through the AMPK-mTOR-TFEB signalling axis. Increased expression of NCoR1 in myeloid cells during the early stage of *Mycobacterium* infection demonstrated its importance for the control of host defence against infection. NCoR1 loss of function impairs the clearance of H37Rv and *M*. *smegmatis* infection in myeloid cells by fine-tuning the AMPK-mTOR signalling axis, which in turn regulates TFEB activity. TFEB autonomously controls autophagic machinery and lysosomal biogenesis, giving *Mtb* a survival advantage in NCoR1 depleted condition. Overexpression of TFEB in NCoR1 depleted macrophages recovered the LC3 and LAMP1 expression and thus cleared the bacterial load. Active mTOR phosphorylates TFEB to inhibit translocation to the nucleus and thus hinder autophagy and lysosome biogenesis [21]. These reports were found to be consistent with our observations. Moreover, pharmacological inhibition of mTOR by rapamycin/Torin1 and activation of AMPK with metformin restored autophagy and consequently protected the macrophages from *Mtb* infection. Overall, our study showed that NCoR1 can influence *Mtb* survival in myeloid cells via modulating the auto-phagolysosomal machinery. NCoR1 has also been shown to regulate diverse biological functions [24]. Our and other previous reports have shown the role of NCoR1 in controlling anti-inflammatory and tolerogenic phenotype in macrophages and DCs, respectively [40,67]. However, any evidence for NCoR1 in regulating autophagy and lysosome biosynthesis processes and *Mtb* survival in host cells has not been reported. These findings gave strong indication that NCoR1 levels are crucial at early stages of *Mtb* infection in myeloid cells to protect from pathology. Among myeloid cells, DCs are also required to activate the systematic immune system to protect microbial insult [66,68]. cDC1 showed peak expression of TFEB earlier as compared to mo-MΦ upon *Mtb* infection that supports the urgency of TFEB mediated induction of autophagy process to control *Mtb* infection. Mechanistically, we demonstrated that NCoR1 regulates TFEB, and its depletion diminishes the auto-phagolysosome formation in cells, making them more susceptible to *Mtb* pathogenesis. To gain a global insight, we looked into the transcriptomics data of *Mtb*-infected NCoR1 KD cells and found key autophagy inducing genes like ATG13, ATG3, and ATG5 to be down-regulated. These ATGs are well reported to drive the autophagy induction process through optimal auto-phagosome maturation and LC3 lipidation [69–71]. We are hypothesising that NCoR1 might provide functional stoichiometry to autophagy machinery proteins in myeloid cells which is important to control *Mycobacterium* pathogenesis. On the other side, NCoR1 has been widely reported to regulate metabolic processes including fatty acid and lipid oxidation, mitochondrial respiration, and ATP synthesis [26,40,67]. We observed an increased mitochondrial ATP as well as total cellular ATP in *M*. *smegmatis-*infected mo-MΦ. *Mycobacterium* mediated changes in host cell oxidative phosphorylation has been shown to be critical for their survival fitness [72–74]. Several reports have shown various evidences where *Mycobacterium* can manipulate cellular energy homeostasis by regulating AMPK-mTOR signalling to its own proliferation and survival [75,76]. Cellular energetic state is tightly monitored by AMPK-mTOR pathway to maintain homeostasis. AMP/ATP ratio sensed by AMPK in turn modulates the mTOR activity to operate optimum autophagic flux, hence refuelling required energy in cellular stress conditions [16,65,77]. As NCoR1 depletion is known to regulate energy homeostasis even without any infection, we performed starvation and heat-killed *Mtb* treatment experiments and found NCoR1 KD cells to have decreased autophagy process, suggesting the role of NCoR1 protein as an important component for the regulation of autophagy machinery. In addition, antimycin-A treatment reduced the p-mTOR levels and increased the AMPK activity, which in turn decreased the *Mtb* survival with the recovery of TFEB and LC3 levels. These results confirmed our observation that NCoR1 fine-tunes the AMPK-mTOR pathway by maintaining an optimal cellular energy state to control the intracellular *Mtb* survival. This mechanistic insight provided us 2 possible roles of NCoR1, first is the interaction with TFEB for its functionality or stability, and second is the regulation of AMPK-mTOR signalling. In conclusion, we revealed here a direct role of NCoR1 in controlling *Mtb* survival by maintaining optimum auto-phagolysosomal process. Furthermore, our results also revealed a clinically significant correlation of NCoR1 expression during active TB infection, which is restored after 12 months of TB treatment, showing a clear link between them. Similar findings were made in *Mtb*-infected PBMCs, where early infection showed increased NCoR1 expression, which thereafter decreased at 24 h. Therapeutically, NCoR1 might be a potential candidate for host-directed therapy.

## Materials and methods

### Ethics statement

Human primary PBMCs used for the experiment were isolated from 10 ml of venous blood isolated from 4 adult healthy control donors at the Institute of Life Sciences, Bhubaneswar, Odisha, India, with necessary ethical approval. The anonymised details of the individuals are provided in Table C in **S3 Table**. The Ethics Committee of the Institute of Life Sciences granted ethical permission for human subject PBMC samples. All procedures were fully compliant with the declaration of Helsinki 2013: the ethical principles for medical research involving human subjects. Before PBMCs were collected for ex vivo investigations as well as comprehensive confocal and qRT-PCR analyses (representative **Fig 1D**, **1E,** and **S1A**), the individuals gave written informed consent, and all the subject data was anonymised. The respective ethics statement for curated publicly available RNA-seq datasets used in representative **Fig 1A** and **1B** are available in the respective publications mentioned in Tables A and B in S3 Table.

### Generation of stable NCoR1 KD in THP-1 human monocytes and cDC1 murine DCs

Lentiviral vector pLKO.1 (Sigma) containing NCoR1-specific shRNAs or control shRNA were used to develop stable NCoR1 KD and corresponding control THP-1 cells. Viral particles containing shRNA and packaging plasmids were generated using CalPhos Mammalian Transfection Kit (Takara, 631312) according to the previously used protocol [40]. After transfection, viral particles were concentrated at 50,000 × g in an ultracentrifuge at 16°C for 2 h. Then, THP-1 cells were transduced with concentrated virus particles and cultured for 72 h in presence of 8 μg/ml of polybrene. Transduced THP-1 cells were then selected using puromycin containing culture media for an additional 2 weeks. The efficiency of NCoR1 KD was quantified using NCoR1 gene-specific primers by qRT-PCR. We employed the CD8^+^ cDC1 mutu-cDC1 line developed by the research group of Prof. Hans Acha-Orbea. These DC lines have been thoroughly studied and compared to primary CD8^+^ cDC1 DCs, and researchers found that they completely mirror immature CD8^+^ DCs that were isolated from C57BL/6 mice’s spleen in ex vivo studies. Control and stable NCoR1 KD cDC1 were directly used for experiments which were characterised previously [39,40,78].

### Cell culture

NCoR1 KD THP-1 cells and NCoR1 KD cDC1 were cultured in RPMI 1640 (Gibco Laboratories) and IMDM (Gibco Laboratories), respectively, supplemented with 10% FBS (Gibco Laboratories). Cells were maintained with their respective controls at a cell density of 2.0 to 10.0 × 10^5^ cells per ml with puromycin (1 μg/ml) at 37°C in a humidified, 5% CO_2_ atmosphere and the KD was confirmed before performing each experiment. The DCs were raised in IMDM-glutamax (GIBCO) that had been buffered with NaHCO_3_ and supplemented with 8% to 10% heat-inactivated FCS (to test for endotoxin toxicity toward DC cultures), 10 mM HEPES (GIBCO 15630), 50 μm β-Mercaptoethanol (GIBCO 31350), 50 U/ml of penicillin, and 50 g/ml of streptomycin (GIBCO 15070). The cells were kept at 37°C in an incubator that was humidified and contained 5% CO_2_. These DCs were separated after a brief incubation at 37°C in a non-enzymatic cell dissociation solution containing 5 mM EDTA (5 mM EDTA in 20 mM HEPES-PBS).

cDC1 cells were seeded in 96-well plates at a density of 1.0 × 10^4^ cells per well or in 6-well plates at a density of 2.5 × 10^6^ cells per well prior to infection, while THP-1 cells were being differentiated with PMA (Sigma, P1269) at a concentration of 20 ng/ml for 17 h prior to infection. THP-1 cells were also plated with the same number of cells as cDC1.

### Bacterial culture

*Mycobacterium* sp. were grown mid-log phase on selective media Middlebrook 7H9 broth (BD Difco, Becton Dickinson) supplemented with 10% ADC (Becton Dickinson), 0.4% Glycerol and 0.05% Tween-80. mCherry-tagged and GFP-tagged *M*. *tuberculosis* and *M*. *smegmatis* were used for microscopy and FACS experiments.

### Infection with *Mycobacterium* species

For infection, *Mycobacterium tuberculosis* (H37Rv strain) or *Mycobacterium smegmatis* single-cell suspensions were prepared and opsonized in antibiotic-free RPMI or IMDM media [9]. Bacteria were quantified by measuring the absorbance at a wavelength of 600 nm (0.6 O.D. corresponds to approximately 100 × 10^6^ bacteria). Cells were infected with an MOI of 1:10 for 2 h followed by 3 times washing by prewarmed FCS-free RPMI media or IMDM media to remove extracellular bacteria. FLAG-tagged TFEB and FLAG-tagged NCoR1 were expressed in NCoR1 KD THP-1 cells by electroporation (Neon Transfection System 100 μl Kit, MPK10025) for 48 h followed by differentiation and infection as explained above.

### PBMCs

For validating the results in primary human macrophages, 10 ml venous blood was obtained from healthy donors in EDTA tubes followed by PBMC separation using lymphoprep (STEMCELL Technologies, 07801/07811). Cells were counted using trypan blue stain using a haemocytometer and 1.0 × 10^6^ cells were plated in 12-well plates. Before infection, the cells (monocytes) were differentiated into macrophages using 20 ng/ml Human GM-CSF (Prospecbio, CYT-221) for 5 days.

### Phagocytosis assay

Cells were incubated with either *M*. *smegmatis*-GFP at an MOI of 10 or 1.0 μm yellow-green polystyrene latex microbeads (Sigma Aldrich, L4655) in the ratio of 10 particles per cell. Incubation conditions included 37°C and 5% CO_2_. *M*. *smegmatis*-GFP or latex beads phagocytosed cells were washed twice with 1× PBS before harvesting, i.e., 10 min, 30 min, and 60 min of incubation, and then they were stained for 10 min at 4°C using Live Dead Fixable Violet Dead Cell Stain Kit (Thermo Fisher Scientific, L34955) [43]. After washing, cells were resuspended in FACS buffer and acquired on BD LSR Fortessa Cell Analyzer (BD Biosciences). Acquired data was analysed using FlowJo-X software (Treestar). In parallel, CFU assay was performed for phagocytosis assay. Infected cells were collected at 10 min, 30 min, and 60 min intervals and lysed in 100 μl of 0.06% of SDS for 10 min at room temperature. Lysates were diluted by 7H9 broth in the ratio 1:10, 1:100, 1:1,000, and 1:10,000 and plated separately in duplicate sets on 7H11 agar plates supplemented with OADC (Becton Dickinson) and 0.5% glycerol and counted on day 21.

### Generation of myeloid-specific NCoR1 knockout mice (NCoR1^MyeKO^)

The CD11c-specific NCoR1 KO homozygous mice were bred with the LysM cre FVB mice to obtain heterozygotes in the F1 generation. Further, the F1 mice were backcrossed with the NCoR1 KO Cd11c-Cre homozygous KO parent to obtain F2. Approximately 25% of the F2 mice showed NCoR1-loxP, CD11c-Cre, and LysM-Cre. These 25% of F2 were self-crossed to obtain F3. F3 generation thus obtained were having 25% of control mice exhibiting only NCoR1-loxP sites and 50% were showing both CD11c-Cre, LysM-Cre along with NCoR1-loxP. Further, to obtain proper inbred genotype, crossing was continued till 10 generations to select experimental mice. Selections were strongly adhered to the mouse genotyping results, added in (**S2M Fig**). LysM-cre mice were chosen due to their high fecundity rate. Since obtaining a pure homozygous breed takes at least 10 crosses [79]. Moreover, crossing C57BL/6 with FVB strains has no profound effect on systemic cytokine levels [80]. Adult mice of 6 to 8 weeks of age of both sexes were used indiscriminately. All the animal experiments were performed after getting due approval from the institutional animal ethics committee.

### BMDM

Mice between the age of 6 to 8 weeks old were humanely euthanized by CO_2_ asphyxiation, and their tibias and femurs were taken for experiments. Bone marrow cells were extracted using RPMI-1640 media supplemented with 10% FCS. In 6-well plates, the cells were seeded at a density of 1.0 × 10^6^ cells/ml, and they were cultured with M-CSF at a concentration of 20 ng/ml for a period of 5 days. Following that, these cells were subsequently infected with H37Rv at a number of infection (MOI) of 1:10, as mentioned earlier, and more experiments were carried out further down the line.

### Peritoneal macrophages

Mice 6 to 8 weeks of age were humanely euthanized by CO_2_ asphyxiation and were dissected out keeping the peritoneal membrane intact. Approximately 5 ml of RPMI-1640 media was injected into the peritoneal cavity. The cells were collected after whirling the cavity for 5 min.

### CFU determination *Mtb*-infected cells

H37Rv post-infected cells at different time points were collected and lysed in 100 μl of 0.06% of SDS for 10 min at room temperature. Lysates were diluted by 7H9 broth in the ratio 1:10, 1:100, 1:1,000, and 1:10,000 and plated separately in duplicate sets on 7H11 agar plates supplemented with OADC (Becton Dickinson) and 0.5% glycerol.

### Intranasal infection of mice with *Mtb*

Before infection, culture of *Mtb*-GFP (H37Rv) bacilli at logarithmic phase (OD_600_ = 0.5–0.6) was aspirated using a 30-gauge needle for 15 to 20 times in order to prevent clumping. To infect mice, anaesthetized animals were given an inoculation of 20 μl containing 10^5^ live bacilli through an intranasal route. Day 1 post infection, the inoculum dose was tracked and controlled by analysing the bacterial load that was present in the lungs of infected mice (10^4^/lung) [45].

### Organ processing

Control (*Mtb*), NCoR1^fl/fl^ (*Mtb*-GFP), and NCoR1^MyeKO^ (*Mtb*-GFP) mice were humanely euthanized and dissected to collect lung and spleen. Tissues were processed for flow cytometry, CFU, histology, and bioplex. Histology samples were kept in 10% formalin and rest were treated with 0.5mg/ml collagenase A (10103578001, Sigma) in RPMI (10% FBS) and incubated for 20 min at 37°C. For single cell suspension, they were RBC lysed and filtered with a 70 μm filter. Samples were then taken for flow, CFU, and bioplex.

### CFU determination in organs

At a variety of post infection time points, i.e., day 7 and day 21, the levels of bacterial load in the lung and spleen of *Mtb*-infected mice were evaluated. In brief, organs from NCoR1^fl/fl^ and NCoR1^MyeKO^ mice that had been humanely euthanized were extracted in an aseptic manner and then homogenised in 0.04% Tween 80. Difco Middlebrook 7H11 Agar (BD Biosciences) plates supplemented with 10% OADC (BD Biosciences) and 0.5% glycerol were used to grow colonies from serial dilutions, which were cultured at 37°C for 21 days before counting.

### FACS from organ

For flow, single-cell suspensions were first stained with live/dead Zombie UV fixable dye (1:1,000 diluted in 1× PBS) for 30 min at RT (as per the manufacturer’s instruction) followed by washing with FACS buffer (3% FBS in 1× PBS). Cells were then stained with a combination of fluorochrome conjugated anti-mouse antibodies (according to the different multicolour group panel) in the FACS buffer for 45 min on ice. The panel comprised of CD45 (BUV496, BD, 423108), CD11c (ef450, ebioscience, 48-0114-82), Ly6C (BV711, biolegend, 128037), Ly6G (PerCP-Cy5.5, biolegend, 127616), MHCII (PerCP-eFlour710, ebioscience, 46-5320-82), Siglec F (APC, biolegend, 155507), F4/80 (AF700, biolegend, 123130), CD11b (APC-Cy7, tonbo, 25-01120U100), CD3 (PeCy5, tonbo, 55-0031-U100), CD8a (APC, tonbo, 20-1886-U100), CD4 (AF700, ebioscience, 56-0042-82), CD19 (APCeF780, ebioscience, 56-0042-82), and CD44 (eF450, ebioscience, 48-0441-82).

Thereafter, cells were washed, fixed in 2% PFA, and acquired on a Cytek Aurora (5 laser and 64 fluorescent detectors). Unmixing was performed using SpectroFlo version (Cytek) software. Auto-fluorescence was removed from the inbuilt software. Gating for *Mtb*-GFP positive infection was in accordance with *Mtb* infected un-tagged negative tissue samples. Further, FMO controls were used to demarcate specific myeloid and lymphoid populations in lungs and spleen. Data were analysed with FlowJo version 10 and compiled in Prism software 8.0.2.

### FACS from cell line

For the flow cytometric analyses of NCoR1 KD THP-1 differentiated macrophages and NCoR1 KD cDC1, post-infected cells were dissociated from plates according to their corresponding time point and washed 3 times with FACS buffer (3% FCS in 1× PBS, 5 mM EDTA). After washing, the cells were acquired for *Mtb* load analysis on Aria II Cell Sorter (BD Biosciences). The acquired data was analysed using FlowJo-X software (Treestar).

### Bio-plex assay

Using a 23-plex mouse Cytokine Panel (M60009RDPD, Biorad) and a Bio-plex (multiplex ELISA-Luminex) reader, cytokines were analysed in *Mtb*-infected lung homogenates from NCoR1^fl/fl^ and NCoR1^MyeKO^ mice, according to the recommended protocol. Prior to beginning the process of quantifying cytokines in the lung tissue samples, a commercial BCA test was done as recommended by Thermo Scientific to normalise the amounts of total protein in the lung lysate samples [81–83].

### Tissue immunofluorescence

Triple immunofluorescence was performed in paraffin embedded murine tissue sections. The lung tissues were dewaxed in xylene for 5 min with 2 changes, followed by an acetone dip for 2 to 3 min followed by rehydration by sequential grades of alcohol for 5 min each. Antigen retrieval was done with proteinase-K solution (20 μg/ml) in 1× PBST (1× PBS + 0.05% Tween-20) followed by 3 washes in 1× PBS. The dewaxed sections were blocked by incubation with a blocking buffer (3% goat serum + 3% BSA in 1× PBST) for 1 h in dark at RT. Primary antibody staining for NCoR1 (1:400), TFEB (1:400), CD11c (1:400), and F4/80 (1:400) was done in 3% BSA in 1× PBST kept in a humidified chamber for 2 h in dark at RT. Subsequent secondary Ab (1:800) incubation was performed with the same buffer composition in a non-humidified chamber for 1 h in dark at RT. Sequential staining was performed with different combinations (CD11c and NCoR1 and F4/80 and NCoR1) followed by TFEB. Finally, the tissue sections were mounted with antifade-mounting media and observed under a microscope.

### Lung inflammation histopathology

For histopathology, lung tissue samples were collected, fixed in 10% neutral-buffered formalin, embedded in paraffin, cut into sections measuring 5 to 6 μm, and stained using the HE stain. The degree of inflammation in the mice’s lungs was assessed as previously mentioned [84]. In order to determine the lung area that was affected, HE-stained lung sections were photographed under a microscope at a magnification of about 5, 10, and 20.

### Inhibitor and inducer treatments

Before harvesting the cells, Bafilomycin A1 (Invivogen, tlrl-baf1) and Rapamycin (Invivogen, tlrl-rap) were added at 100 nM and 20 nM, respectively, for 2 h and 24 h to monitor autophagy flux. In order to determine whether or not the phosphorylation of TFEB at Ser211 was reliant on mTORC activity, we treated Control versus NCoR1 KD THP-1 differentiated *Mtb*-infected cells with another mTOR inhibitor, Torin1, at 250 nM concentration (Selleckchem, S2827) [85]. Following *Mtb* infection, protein expressions were analysed using western blotting. Cells were also treated with 0.125 μm Antimycin-A (Sigma, A8674) prior to H37Rv infection and harvested post infection at 6 h for CFU assay, western blot, and FACS. Cell viability assays of Antimycin-A treated cells were performed with the LIVE/DEAD Fixable Violet Dead Cell Stain Kit (Thermo Fisher Scientific, L34955). Further, metformin (20 nM) treatment was done (Sigma, 317240) to quantitate survival by CFU assay.

### Immunoblotting

Cells were lysed in buffer containing 1% tritonX-100, 0.1% SDS, 50 mM HEPES (pH 7.5), 150 mM NaCl, 100 mM NaF, 10 mM EDTA, 10 mM Na_4_P_2_O_7_, and protease inhibitors (Roche). BCA assay (Pierce BCA Protein Assay Kit, 23225) was used for protein estimation. Cell lysates were separated by SDS-PAGE and transferred onto Nitrocellulose membrane (BIO-RAD, 1620112). Membranes were then probed with primary antibodies of TFEB (Cell Signaling Technology, 4240S), NCoR1 (Abcam, ab24552), LC3 (Cell Signaling Technology, 2775S), Phospho-mTOR (Ser2448) (Cell Signaling Technology, 5536S), mTOR (Cell Signaling Technology, 2972S), Phospho-AMPKα (Cell Signaling Technology, 50081T), AMPKα (Cell Signaling Technology, 5831T), Beclin1 (Cell Signaling Technology, 3495S), ATG12 (Cell Signaling Technology, 4180S), FLAG (Cell Signaling Technology, 14793S), Phospho-TFEB (Ser211) (Cell Signaling Technology, 37681S), LAMP1 (Cell Signaling Technology, 3243S), and β-actin (Cell Signaling Technology, 8457S).

### Immunofluorescence *(*in vitro and ex vivo)

After each time point, cells were washed with 1× PBS and fixed with 4% paraformaldehyde (Sigma) for 20 min, followed by 3 washes with 1× PBS. The cells were permeabilized using 0.2% (w/v) TritonX-100 prepared in 1× PBS for 20 min and blocked with 3% (w/v) BSA and 0.5% Tween20 in 1× PBS for 1 h. After blocking, cells were stained for 2 h at RT with a primary antibody (1:200), washed thoroughly 3 times with 1× PBST (0.5% Tween20 in 1× PBS), then incubated for 45 min at RT with suitable Alexa-Fluor conjugated secondary antibodies (1:500). After incubation, the cells were washed 3 times in 1× PBS and mounted with an antifade-DAPI mounting solution (Invitrogen, P36983) before being examined under a microscope. Primary antibodies used in the study were TFEB (Cell Signaling Technology, 4240S), NCoR1 (Abcam, ab24552), LC3 (Cell Signaling Technology, 2775S), and LAMP1 (Cell Signaling Technology, 3243S).

### RNA extraction and qRT-PCR

RNA was extracted from NCoR1 KD THP-1 cells or PBMCs-derived macrophages using NucleoSpin RNA Plus miniprep kit (Macherey-Nagel 740984.250) as per manufacturer’s manual. Then, cDNA was synthesised using the Transcriptor First-Strand cDNA Synthesis Kit (Roche Applied Science, Indianapolis, Indiana, United States of America). For gene expression analysis, qRT-PCR was performed using Power SYBR Green PCR Master Mix (Invitrogen) on a QuantStudio-6 Flex Real-Time PCR System (The Applied Biosystems). Human and mouse-specific primers used for qRT-PCR are provided in **S4 Table**.

### ATP determination assay

Intracellular ATP was evaluated using an ATP determination kit (Invitrogen, A22066), according to the manufacturer’s instructions. NCoR1 KD and Control THP-1 differentiated macrophages (approximately 10^5^ cells) were infected with *Mycobacterium* (1:10 MOI) and intracellular ATP was extracted at various time intervals. Dissociated cells were pelleted and washed twice with 1× PBS after centrifugation at 12,000 × g for 10 min. After that, 1 ml boiling water (90°C) was added to the cell pellet and vortexed vigorously to extract the cellular ATP. Following the vortex, the lysed cells were centrifuged at 12,000 × g for 5 min at 4°C, with 20 μl of the suspension used for bioluminescence measurement as directed by the kit manufacturer. Serial dilutions of 10 μm ATP solution were used to generate the ATP standard curve.

### Extracellular flux assay (Seahorse)

Mito-Stress Test (Agilent technologies, 103010–100) was performed on a Seahorse XFp extracellular flux analyzer. Control and NCoR1 KD cells were plated on a sterile XFp plate (Agilent technologies, C21119) in triplicates at 2.0 × 10^4^ total cells per well with sequential additions of the following compounds 1 μm oligomycin, 2 μm carbonyl cyanide-4-(trifluoromethoxy) phenylhydrazone (CCCP), and 1 μm rotenone/antimycin-A. All the chemicals were prepared according to the manufacturer’s protocol. Respiratory parameters like basal oxygen consumption rate (OCR), coupled ATP production, maximal respiratory capacity, and spare respiratory capacity were calculated using the WAVE software.

### Library preparation for RNA-seq

Total RNA was extracted from *Mtb*-infected THP-1 differentiated macrophages with RNeasy Plus Mini Kit according to the manufacturer’s instructions. The RNA was quantified by NanoDrop 2000 Spectrophotometer and purity was assessed by a 2100 Bioanalyzer (Agilent Technologies, Waldbronn, Germany). RNA integrity number (RIN) >8, RNA was taken for library preparation. llumina TruSeq Stranded Total RNA Library Prep Human/Mouse/Rat Kit (llumina, San Diego, California, USA) was used for preparation of sequencing library with 1 μg of total RNA for each sample. Total RNA samples were treated with the Ribo-Zero rRNA Removal Kit (llumina, San Diego, California, USA) to deplete bacterial and eukaryotic ribosomal RNA (rRNA). First, Ribo-zero treated RNA was used to synthesise single-stranded cDNA and then second strand was synthesised using DNA Polymerase I and RNase H to produce double-stranded cDNA. Next, the cDNA was fragmented and the cDNA fragments were end repaired by addition of single “A” and then indexing adapters were ligated to each sample. The products were then purified and enriched using PCR with adapter universal primers to generate NGS libraries. Final prepared libraries were then quantified and checked for fragment size using Qubit High sensitivity DNA reagent (Qubit 2.0) followed by TapeStation D1000 ScreenTape (Agilent Technologies). The resulting NGS libraries were then sequenced using the NextSeq550 platform (llumina).

### RNA-seq data curation and processing

From demultiplexed FASTQ files low-quality reads and adapter sequences were removed using Cutadapt. Trimmed files were then aligned using HISAT2 to the human reference genome (GRCh38) with rna-strandness set to RF. The aligned data was quantified using featureCounts and GENCODE human genome (GRCh38) GTF version 33 with -p and -s 2 option enabled. Differential expression analysis performed using DESeq2, genes having p adjusted < 0.05 and log2 fold change < = -0.5849, > = +0.5849 considered for further analysis. Pathway enrichment analysis was performed using the ClusterProfiler R package.

Public datasets downloaded from NCBI SRA for the search term “active tuberculosis” and “healthy control” using RNA expression by next generation sequencing and *Homo sapiens* filter terms [32,33]. The samples were processed using a similar analysis process except p adjusted <0.001 cut off was used, outliers were filtered based on normalised expression PCA and no filtering was done based on fold change values. The curated publicly available RNA-seq datasets used in representative **Fig 1A and 1B** are available in Tables A and B in **S3 Table**.

### Computational and statistical analysis

Details of statistical tests can be found in figure legends and statistics for genomic analysis. Prism version 5 and 8.0.2 (Figs 3 and **S3**) were used for statistical calculations. The bar plot of fold changes in gene expression was generated using the GGPLOT2 R package, pairwise comparison and plotting of tuberculosis treatment data were done using the GGPUBR package. Heat maps were plotted using ComplexHeatmap package and hierarchical clustering was performed using the inbuilt clustering functions.

## Supporting information

S1 Table Differentially expressed genes from RNA-seq Data. (XLSX)

S2 Table Up- and down-regulated pathways from RNA-seq Data. (XLSX)

S3 Table Publicly available Metadata across TB subjects. (XLSX)

S4 Table Resources and primers. (XLSX)

S1 Data Numerical values for all datasets. Figs 1A–1C, 1E–1G, 2B–2C, 2E, 2G–2H, 2J–2M, 2O–2R, 3A–3K, 4A–4C, 4E–4F, 4H, 4J, 4L, 5A–5G, 5I, 5K, 5M, 5N, 6A, 6C–6J, 6L, 6M, **S1A–S1**B, **S2**A–**S2**B, **S2**E, **S2**G–**S2**H, **S2**J, **S2**L–**S2**N, **S2**O, **S4**C, **S4**E–**S4**F, **S4**H, **S5**B, **S5**F, **S5**H, **S5**J, **S5**, **S5**L, **S5**N, **S5**P, **S5**R, **S6**A–**S6**D, **S6**G, **S6**I, and **S6**J.
(XLSX)

S1 Raw Images Uncropped western raw images. Figs 1F, 1G, 2A, 2I, 2N, 4D, 4G, 4I, 5A, 5B, 5D, 5E, 5H, 5J, 5L, 6B, 6H, 6K, **S4**D, **S4**F, **S5**A, **S5**E, **S5**M, **S5**K, **S5**O, and **S6**H.
(PDF)

S1 Fig NCoR1 expression in PBMCs and murine cDC1 DCs. (A) RT-qPCR line graph showing the *NCOR1* transcript kinetics (2 h, 12 h, 24 h, and 48 h) in H37Rv infected human PBMCs (*n* = 3 independent biological repeats). (B) RT-qPCR line graph showing the *Ncor1* transcript kinetics (2 h, 12 h, and 24 h) upon *M*. *smegmatis* infection in cDC1 (*n* = 3 independent biological repeats). *p < 0.05, *p < 0.01, and ***p < 0.001 were considered significant. Data analysis was performed using one-way ANOVA with Tukey’s statistical test. Where *n* represents independent biological replicates. The data underlying this figure are available in S4 Table and S1 Data.
(TIF)

S2 Fig NCoR1 perturbation increases *Mycobacterium* burden in myeloid cells. (A) Bar plot depicting the *NCOR1* transcript expression at 2 h, 6 h, and 24 h of H37Rv infected control and NCoR1 KD human monocytic THP-1 differentiated macrophages (*n* = 3). (B) Scatter plot showing the *M*. *smegmatis* load in control and NCoR1 KD human monocytic THP-1 differentiated macrophages by CFU assay at 24 h post infection (*n* = 4). (C) Flow cytometry dot plot showing the percentage of *M*. *smegmatis* infected control and NCoR1 KD human monocytic THP-1 differentiated macrophages at 6 h post infection (*n* = 3). (D) Flow cytometry histograms showing MFI shifts for the *M*. *smegmatis* infection in control and NCoR1 KD human monocytic THP-1 differentiated macrophages at 6 h post infection (*n* = 3). (E) Bar plot showing the quantification of percent positive cells and MFI shifts for the *M*. *smegmatis* infected control and NCoR1 KD human monocytic THP-1 differentiated macrophages at 6 h post infection (*n* = 3). (F) Flow cytometry plots showing the back gating strategies used in flow cytometry analysis. (G) Bar plot of shRNA3-mediated NCoR1 depletion shown by RT-qPCR (*n* = 3). (H) Scatter plot showing the H37Rv load in control and shRNA3-mediated NCoR1 KD human monocytic THP-1 differentiated macrophages by CFU assay at 24 h (*n* = 6). (I) Flow cytometry contour plot showing the phagocytosis rate of GFP-tagged *M*. *smegmatis* in control and NCoR1 KD human monocytic THP-1 differentiated macrophages at 10 min, 30 min, and 60 min post infection (*n* = 3). (J) Bar plot showing the quantification of phagocytosis rate of *M*. *smegmatis* in control and NCoR1 KD human monocytic THP-1 differentiated macrophages at 10 min, 30 min, and 60 min post infection (*n* = 3). (K, L) Microscopy images and bar plot showing the levels of H37Rv infection in control and shRNA3-mediated NCoR1 KD at 24 h post infection (*n* = 3). (M) PCR results showing genotyping of NCoR1^fl/fl^ and NCoR1^MyeKO^ mice. (N) RT-qPCR depicting transcript levels of NCoR1 in CD11c^+^ cells compared to CD11c^-^ fraction (*n* = 2 mice). (O) Scatter plot showing the *M*. *smegmatis* load in control and NCoR1 KD cDC1 by CFU assay at 6 h post infection (*n* = 6). (P) Microscopy images showing the levels of *M*. *smegmatis* infection in control and NCoR1 KD cDC1 at 6 h post infection (*n* = 3). *p < 0.05, *p < 0.01, and ***p < 0.001 using paired and unpaired two-tailed Student’s *t* test. Where *n* represents independent biological replicates. The data underlying this figure are available in S4 Table and S1 Data.
(TIF)

S3 Fig Gating strategies and cell numbers of myeloid and lymphoid cell types in lung and spleen. (A) Schematic outline depicting the in vivo experimental strategy for *Mtb* infection in mice. (B) Images showing the size of spleens isolated from NCoR1^fl/fl^ and NCoR1^MyeKO^ mice at 21 days post H37Rv infection (*n* = 5 mice). (C) Flow cytometry plots showing the gating strategy used for identification of myeloid cell subtypes in the lung tissues of NCoR1^fl/fl^ and NCoR1^MyeKO^ mice at day 21 post infection. (D) Bar plots depicting the myeloid cell numbers in the lung along with *Mtb* infected ones. (E) Flow cytometry plots showing the gating strategy used to analyse myeloid cell subtypes in the spleen tissues of NCoR1^fl/fl^ and NCoR1^MyeKO^ mice at day 21 post infection. (F) Bar plots demonstrating the myeloid cell numbers in the spleen along with *Mtb* infected ones. (G) Flow cytometry plots showing the gating strategy used to analyse B and T cell subtypes in the splenic tissues of NCoR1^fl/fl^ and NCoR1^MyeKO^ mice at day 21 post infection. (H) Bar plots showing the lymphoid cell numbers in the spleen. *p < 0.05, *p < 0.01, and ***p < 0.001 using unpaired two-tailed Student’s *t* test. Where *n* represents the total number of used mice. The data underlying this figure are available in S1 Data.
(TIF)

S4 Fig NCoR1 controls the autophagy induction in both human and murine myeloid cells upon *Mycobacterium* infection. (A) Flow cytometry contour plots showing the intracellular H37Rv bacterial load in control and NCoR1 KD human monocytic THP-1 differentiated macrophages, with and without bafilomycin treatment (*n* = 3). (B, C) Flow cytometry histograms showing the MFI shifts for the H37Rv infection in control and NCoR1 KD human monocytic THP-1 differentiated macrophages with and without treatment of bafilomycin, bar plots depicting the quantification of the same (*n* = 3). (D) Western blot image showing the NCoR1 and LC3-II:LC3-I protein levels in control and NCoR1 KD cDC1 at different time points upon H37Rv infection (*n* = 3). (E) Bar plot showing densitometric quantification for the NCoR1 and LC3-II:LC3-I levels in control and NCoR1 KD cDC1 at different time points upon H37Rv infection. All protein bands were normalised with β-actin housekeeping control (*n* = 3). (F) Western blot image and corresponding densitometric analysis demonstrating the LC3-II:LC3-I protein levels in control and NCoR1 KD THP-1 differentiated mo-mΦ upon H37Rv infection vs. uninfected (*n* = 3). (G, H). Confocal microscopy and corresponding bar plot demonstrating the colocalization of H37Rv with LC3 protein in the BMDMs from NCoR1^fl/fl^ and NCoR1^MyeKO^ mice at 2 h and 24 h post infection (*n* = 4 mice). *p < 0.05, *p < 0.01, and ***p < 0.001 using paired and unpaired two-tailed Student’s *t* test, where *n* represents independent biological replicates. The data underlying this figure are available in S1 Data. Western blot raw images can be found in S1 Raw Image.
(TIF)

S5 Fig NCoR1 controls the mTOR-TFEB axis to regulate autophagy and lysosome biogenesis in myeloid cells. (A, B) Representative western blot image with corresponding densitometric analysis depicting the TFEB protein kinetics (2 h, 12 h, and 24 h) in the H37Rv infected cDC1. All protein bands were normalised with β-actin housekeeping control (*n* = 3). (C, D) Microscopy images showing the relative levels of NCoR1 and TFEB levels in CD11c+ and F4/80+ H37Rv-GFP infected lung tissue sections compared to uninfected C57BL/6 mice. (E, F) Western blot image and bar plot demonstrating the TFEB level in control and NCoR1 KD cDC1 at different time points upon H37Rv infection (*n* = 3). (G) Confocal microscopy showing NCoR1 and TFEB expression in H37Rv infected BMDMs generated from NCoR1^MyeKO^ and NCoR1^fl/fl^ mice (*n* = 4 mice). (H) Bar plot showing the quantification for NCoR1 and TFEB protein levels from confocal microscopy of H37Rv infected BMDMs generated from NCoR1^MyeKO^ and NCoR1^fl/fl^ mice (*n* = 4 mice). (I, J) Confocal microscopy images and bar plots showing the entrapment of H37Rv with LAMP1 protein in control and NCoR1 KD human monocytic THP1 differentiated macrophages at different time points (*n* = 3). (K) Western blot image showing the protein levels of NCoR1, TFEB, and LC3-II:LC3-I in starved and fed condition in control and NCoR1 KD human monocytic THP-1 differentiated macrophages (*n* = 3). (L) Bar plot showing densitometric quantification of NCoR1, TFEB, and LC3-II:LC3-I western bands in starved and fed condition in control and NCoR1 KD human monocytic THP-1 differentiated macrophages. All bands were normalised with β-actin as housekeeping control (*n* = 3). (M) Western blot image showing NCoR1 and LC3-II:LC3-I protein levels in control and NCoR1 KD human monocytic THP-1 differentiated macrophages treated with heat killed H37Rv at different time points (*n* = 3). (N) Bar plot depicting densitometric quantification of NCoR1 and LC3-II:LC3-I in control and NCoR1 KD human monocytic THP-1 differentiated macrophages treated with heat killed H37Rv at different time points. All bands were normalised with β-actin as housekeeping control (*n* = 3). (O) Western blot representative image depicting the p-mTOR, mTOR, and p-TFEB, TFEB levels in H37Rv infected control and NCoR1 KD human monocytic THP-1 differentiated macrophages at 2 h and 24 h post infection, with and without Torin1 treatment (*n* = 3). (P) Bar plot depicting the densitometric quantification of normalised p-mTOR and p-TFEB protein bands. The p-mTOR and p-TFEB levels were normalised first with their respective total protein levels and then with housekeeping control β-actin, with and without Torin1 (*n* = 3). (Q) FACS analysis demonstrating the MFI shifts for H37Rv infection in control and NCoR1 KD human monocytic THP-1 differentiated macrophages at 24 h of infection with and without treatment of rapamycin (*n* = 3). (R) Bar plot demonstrating the MFI shift quantification of H37Rv infection in control and NCoR1 KD human monocytic THP-1 differentiated macrophages at 24 h of infection with and without treatment of rapamycin (*n* = 3). *p < 0.05, *p < 0.01, and ***p < 0.001 using paired and unpaired two-tailed Student’s *t* test. Where *n* represents independent biological replicates. The data underlying this figure are available in S1 Data. Western blot raw images can be found in S1 Raw Image.
(TIF)

S6 Fig NCoR1 regulates mTOR activity by fine-tuning cellular ATP-AMPK level. (A) Pathway enrichment analysis showing the top pathways for the list of down-regulated genes found in RNA-seq data of NCoR1 KD human monocytic THP-1 differentiated macrophages as compared to control cells at 24 h post H37Rv infection (*n* = 3). (B) Heat map depicting differentially expressed genes of oxidative phosphorylation in post-infected Control and NCoR1 KD cells (*n* = 3). (C) Bar graph showing the intracellular ATP level in control and NCoR1 KD human monocytic THP-1 differentiated macrophages upon *M*. *smegmatis* infection at 6 h time point with and without antimycin A treatment (*n* = 3). (D) Scatter plot demonstrating the *M*. *smegmatis* bacterial load in control and NCoR1 KD human monocytic THP-1 differentiated macrophages at 6 h time point by CFU assay with and without antimycin A treatment (*n* = 3). (E) Flow cytometry contour plots depicting the GFP-tagged *M*. *smegmatis* infection in control and NCoR1 KD human monocytic THP-1 differentiated macrophages at 6 h with and without antimycin A treatment (*n* = 3). (F) Flow cytometry histogram plots showing the MFI shifts for *M*. *smegmatis* infection load in control and NCoR1 KD human monocytic THP-1 differentiated macrophages at 6 h with and without antimycin A treatment (*n* = 3). (G) Bar plots depicting the quantitation of percent positive infected cells and corresponding MFI shifts in flow cytometry analysis of *M*. *smegmatis* infection in control and NCoR1 KD human monocytic THP-1 differentiated macrophages at 6 h with and without antimycin A treatment (*n* = 3). (H) Western blot representative image demonstrating the p-AMPKα, p-mTOR, TFEB, and LC3 protein levels in *M*. *smegmatis* infected control and NCoR1 KD human monocytic THP-1 differentiated macrophages with and without antimycin A treatment (*n* = 3). (I) Bar plots demonstrating the quantification of p-AMPKα, p-mTOR, TFEB, and LC3 western blot bands in *M*. *smegmatis* infected control and NCoR1 KD human monocytic THP-1 differentiated macrophages with and without antimycin A treatment. All the phosphorylated proteins were normalised with their totals first, followed by β-actin housekeeping control (*n* = 3). (J) Scatter plot showing percent viability upon antimycin A treatment by FACS at 6 h (*n* = 6). *p* < 0.05, *p < 0.01, and ***p < 0.001 using two-tailed paired Student’s *t* test. Where *n* represents independent biological replicates. The data underlying this figure are available in S1 and S2 Tables and S1 Data. Western blot raw images can be found in S1 Raw Image.
(TIF)

S7 Fig Graphical abstract. “Created with BioRender.com.”
(PDF)

## Abbreviations

AMPK, AMP: activated protein kinase
ATP: adenosine triphosphate
BMDM: bone marrow-derived macrophage
DC: dendritic cell
DEG: differentially expressed gene
HE: haematoxylin and eosin
KD: knockdown
KO: knock out
MFI: mean fluorescence intensity
Mtb: *Mycobacterium tuberculosis*
OCR: oxygen consumption rate
PBMC: peripheral blood mononuclear cell
PRR: pathogen recognition receptor
RIN: RNA integrity number
TB: tuberculosis
TFEB: transcription factor EB
