# Supplementary material for: NCoR1 controls Mycobacterium tuberculosis growth in myeloid cells by regulating the AMPK-mTOR-TFEB axis
Source: PLoS Biol. 2023 Aug 17;21(8):e3002231. doi: 10.1371/journal.pbio.3002231 (PMC10465006; doi:10.1371/journal.pbio.3002231)

Main Figure Western Blot Raw Images - 1

Figure 1F

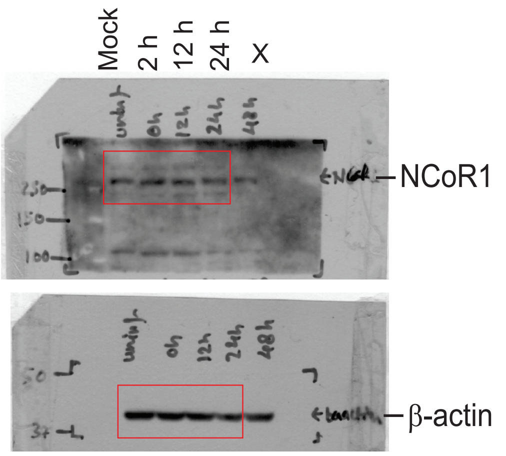

Figure 1G

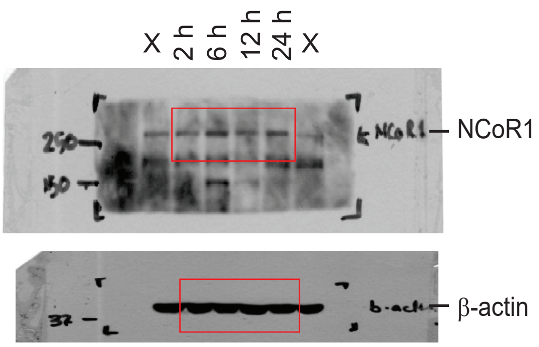

Figure 2A

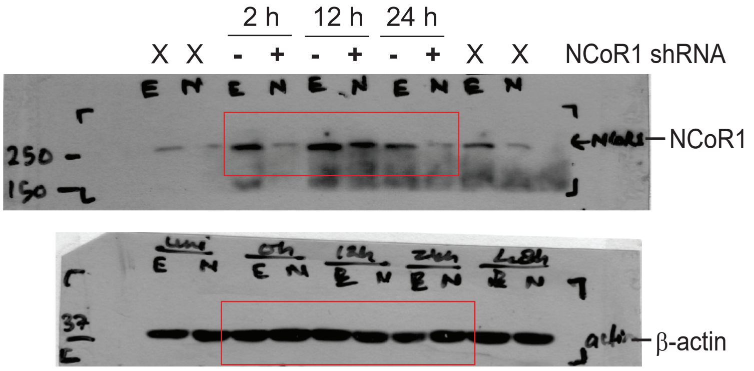

Figure 2I

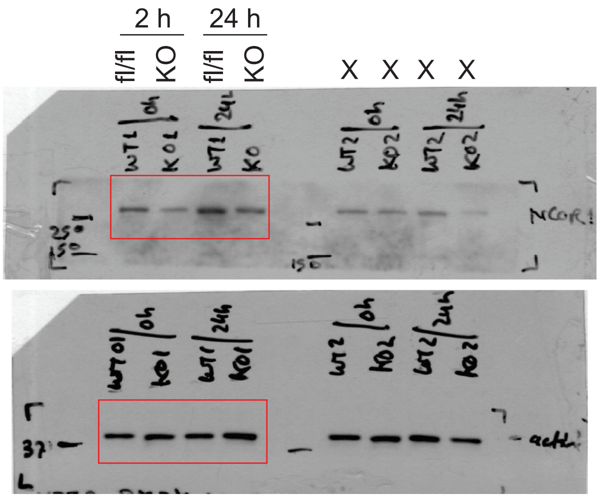

Figure 2N

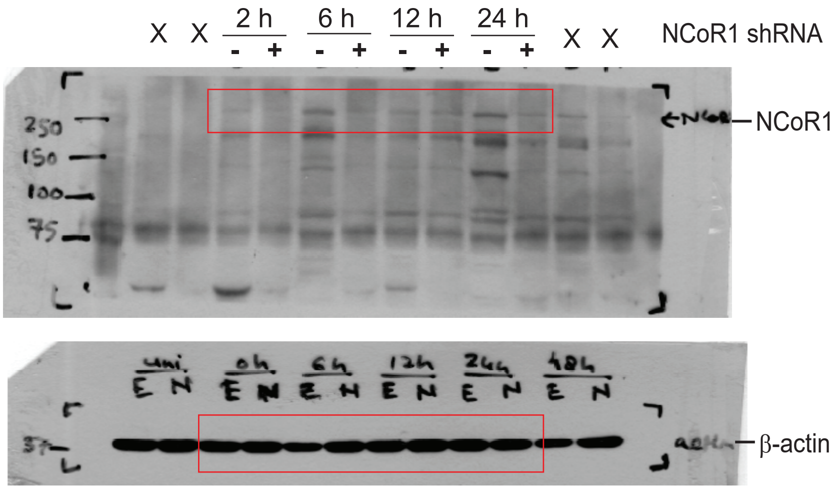

Figure 4D

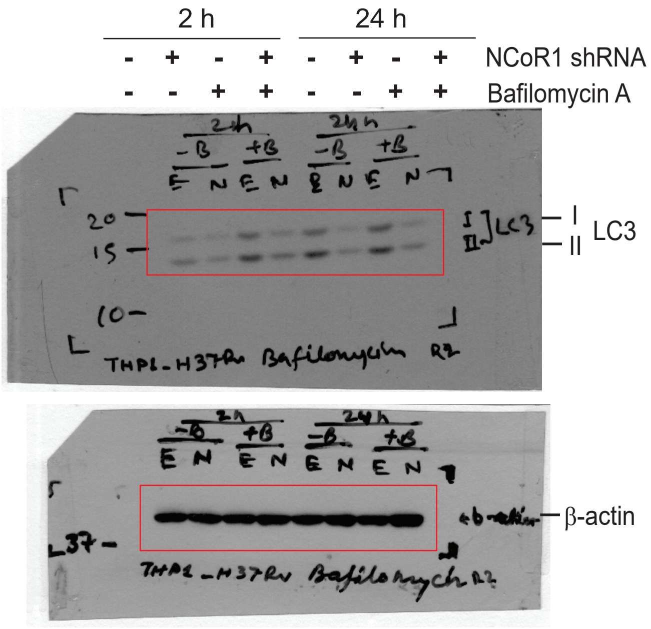

Figure 4G

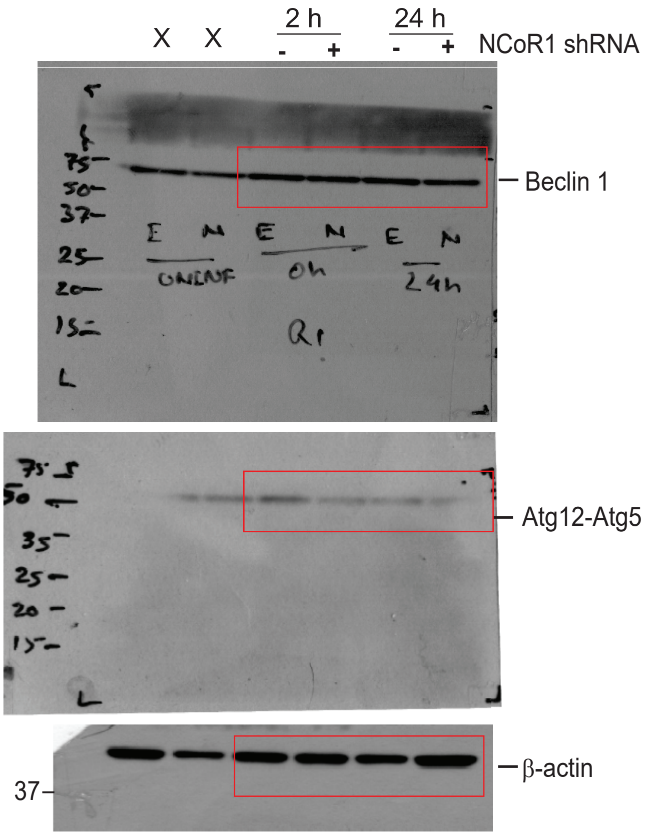

Main Figure Western Blot Raw Images - 2

Figure 4I

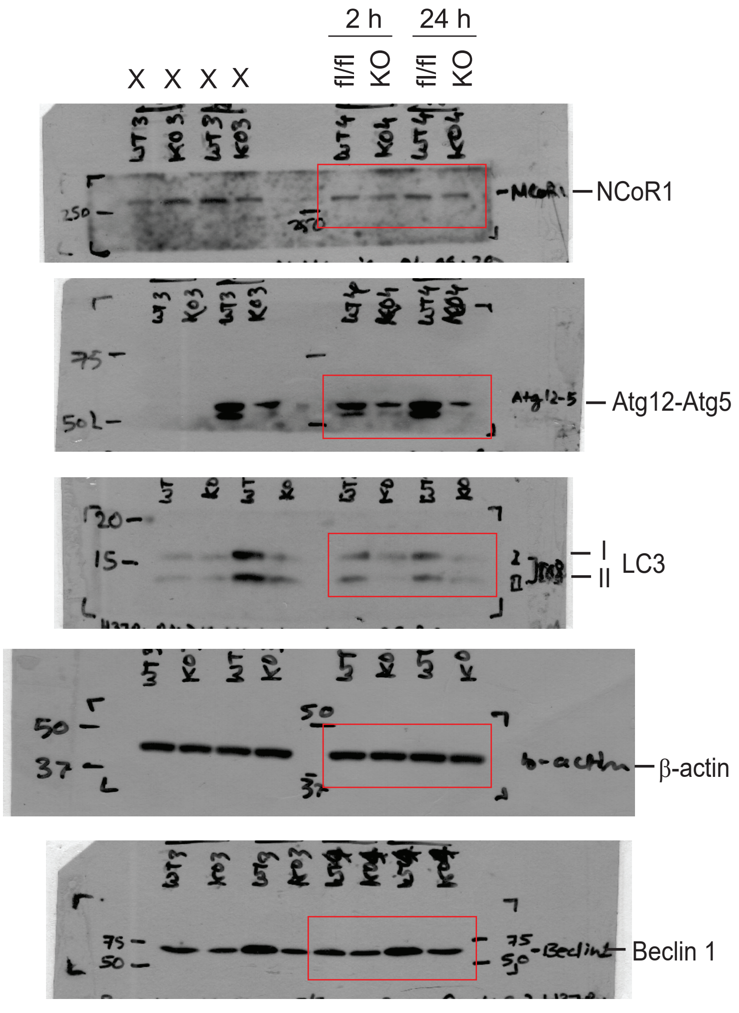

Figure 5A

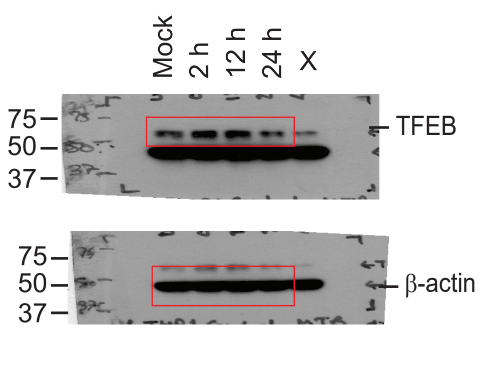

Figure 5B

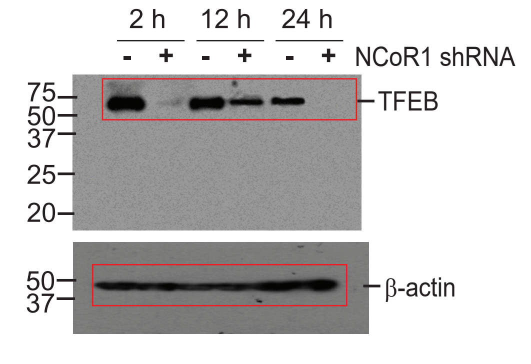

Figure 5D

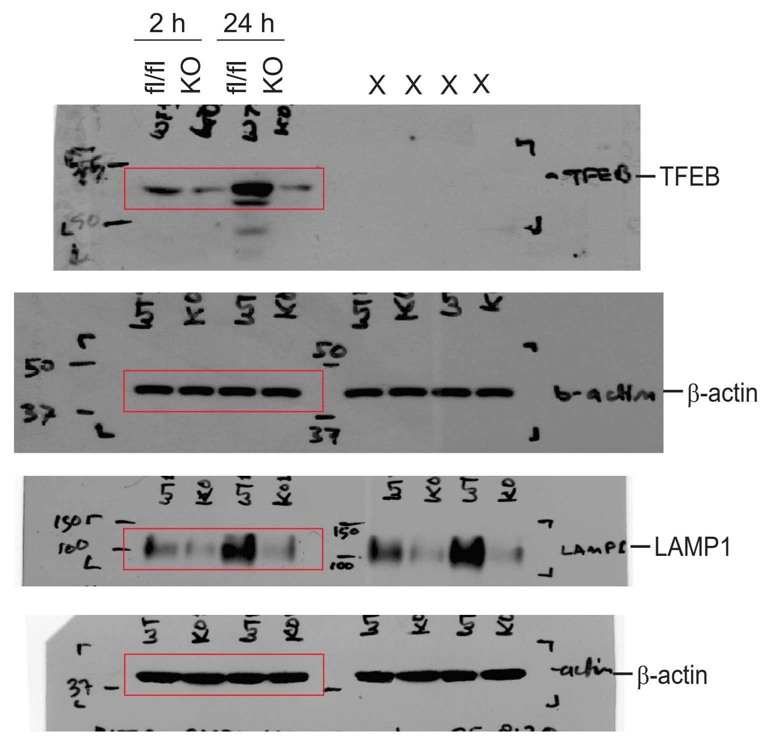

Figure 5E

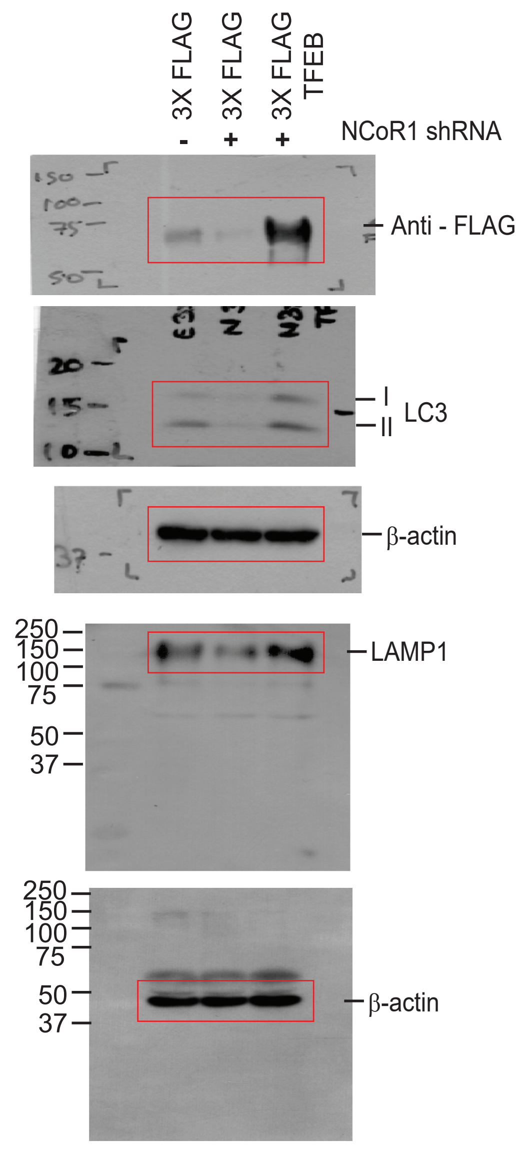

Main Figure Western Blot Raw Images - 3

Figure 5H

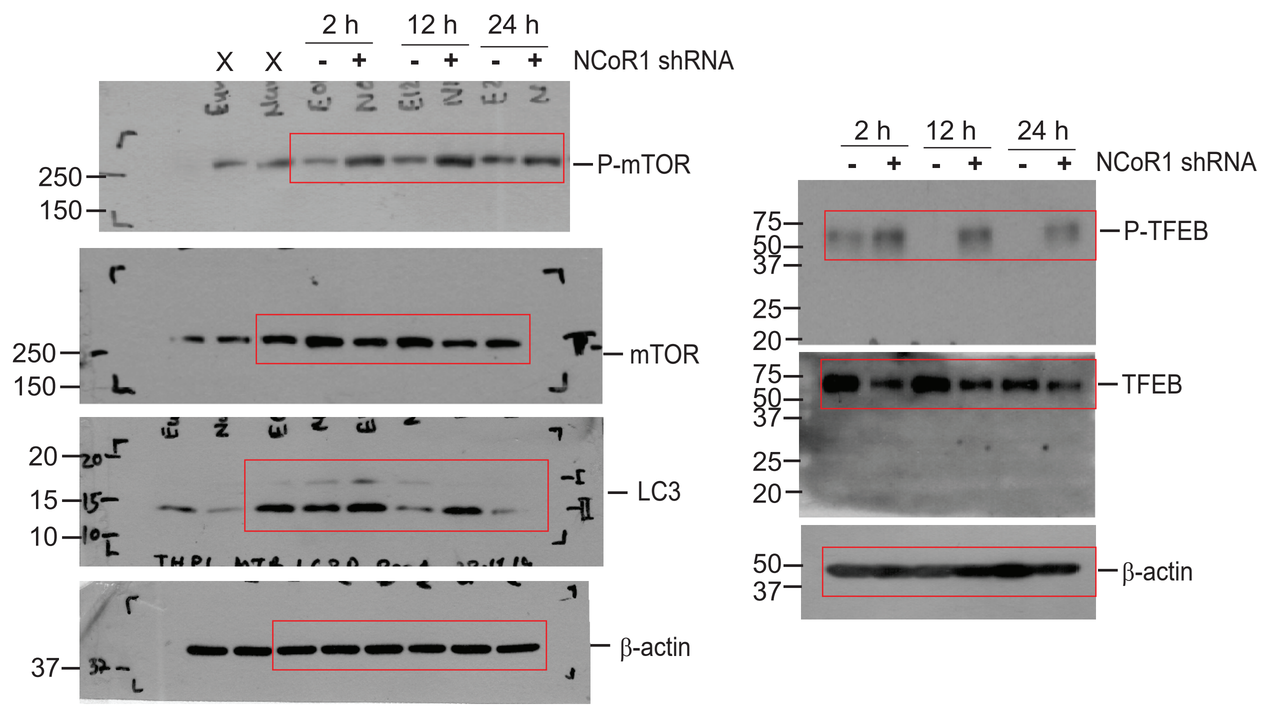

Figure 5J

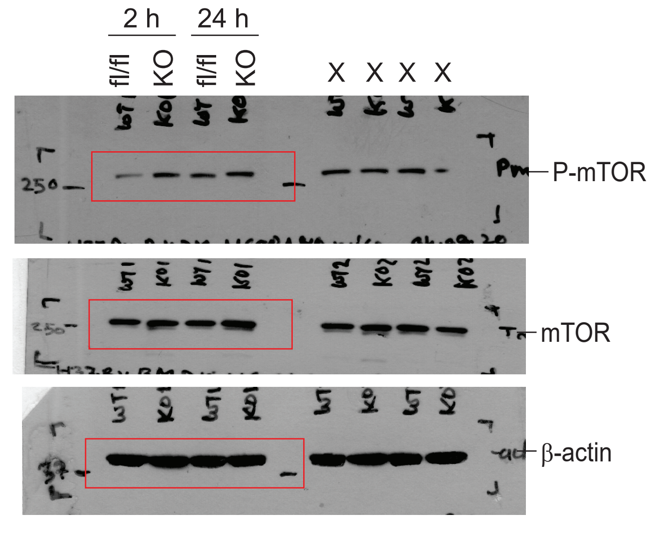

Figure 5L

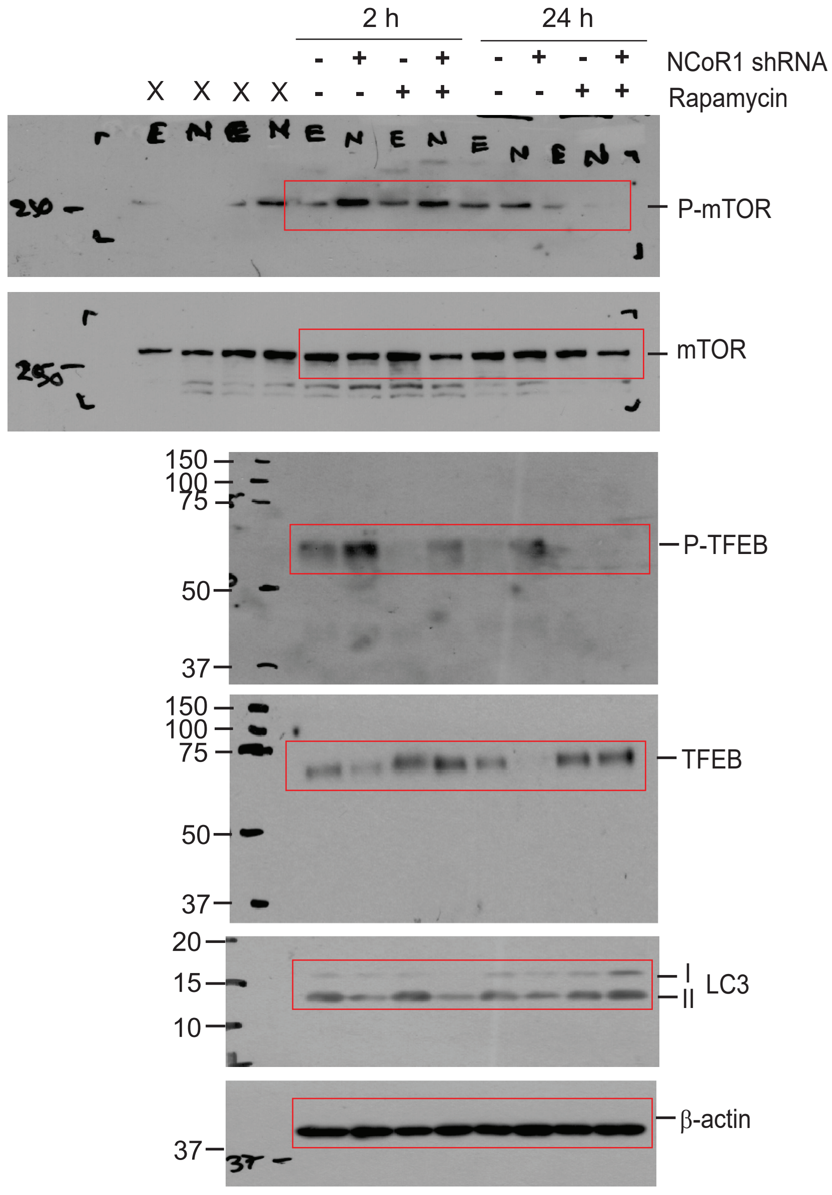

Main Figure Western Blot Raw Images - 4

Figure 6B

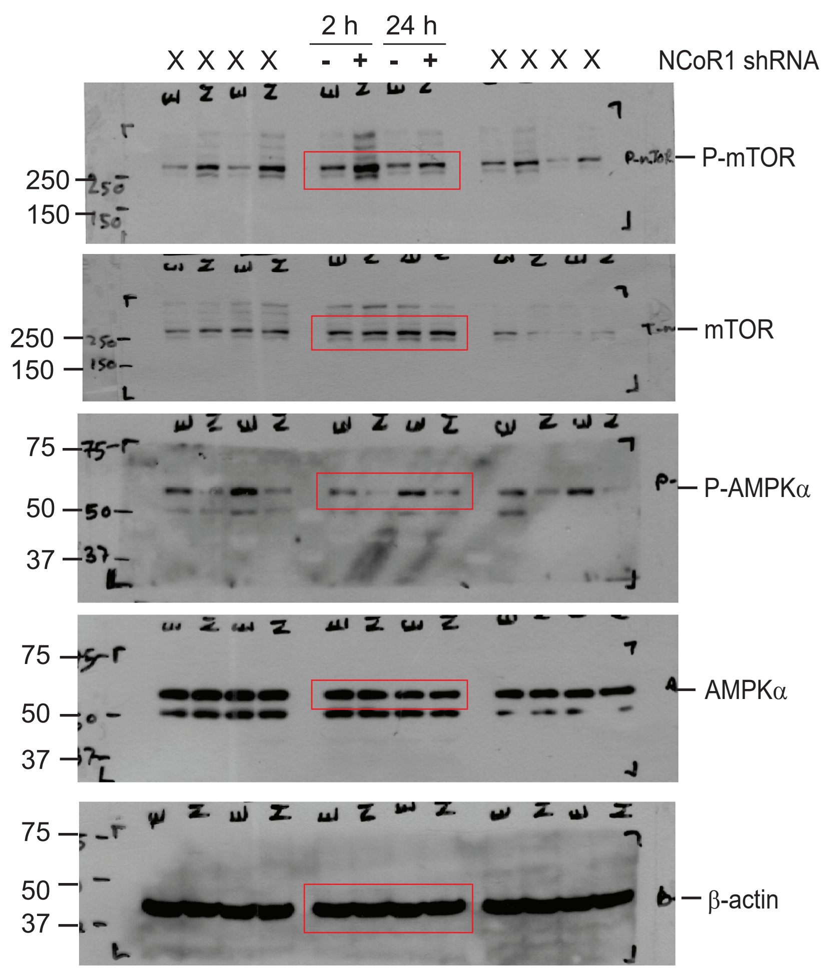

Figure 6K

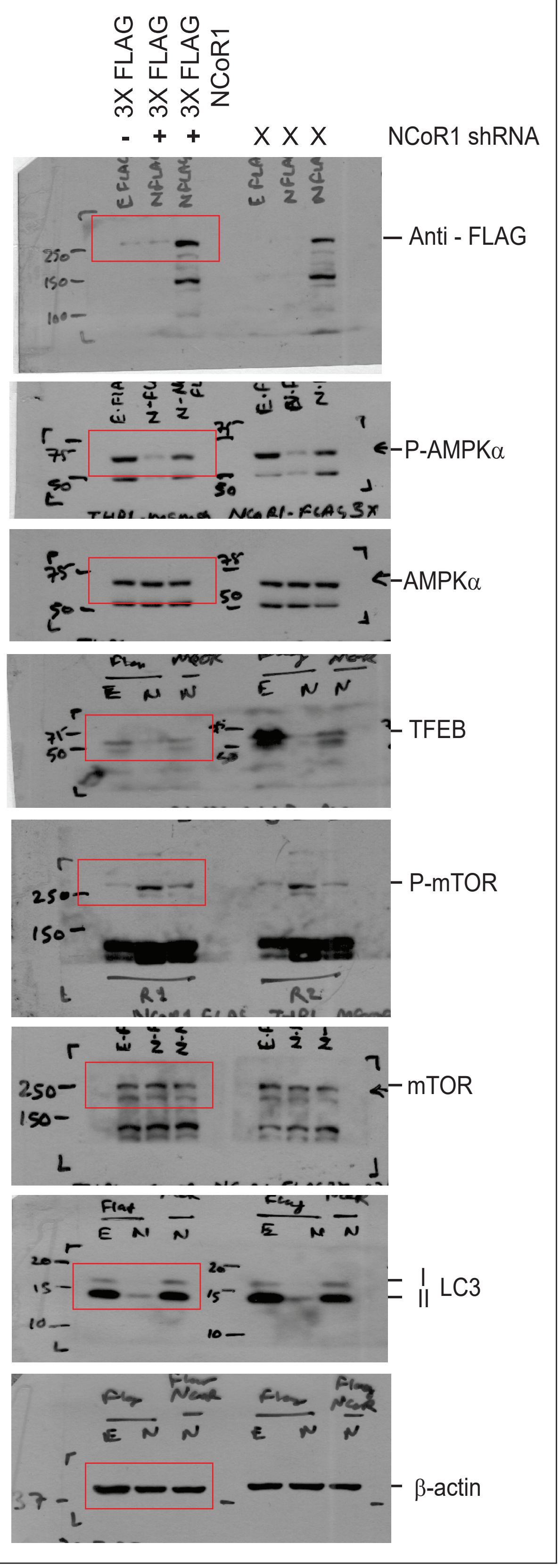

Figure 6H

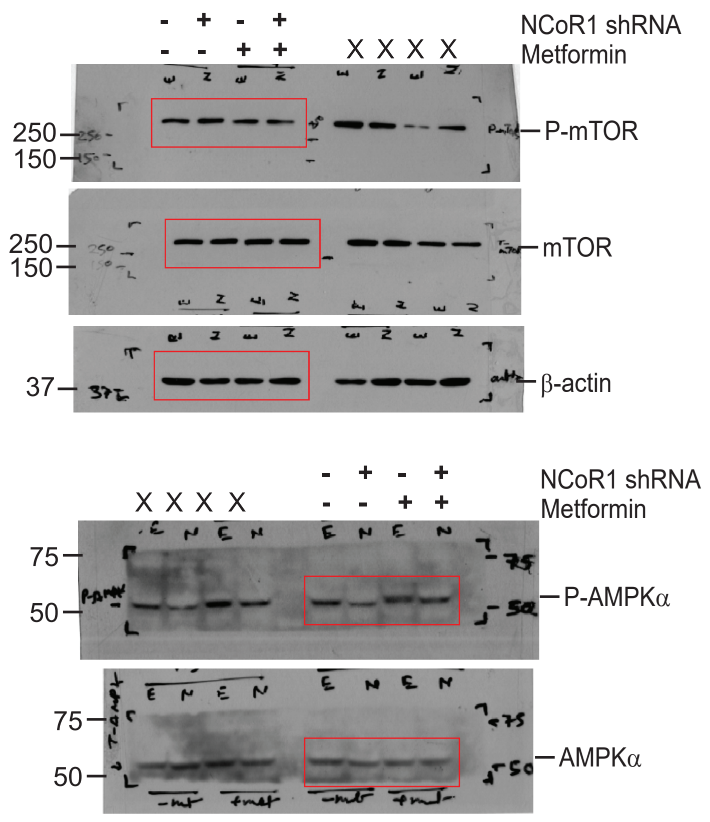

Supplementary Figure Western Blot Raw Images - 1

Supplementary Fig 4D

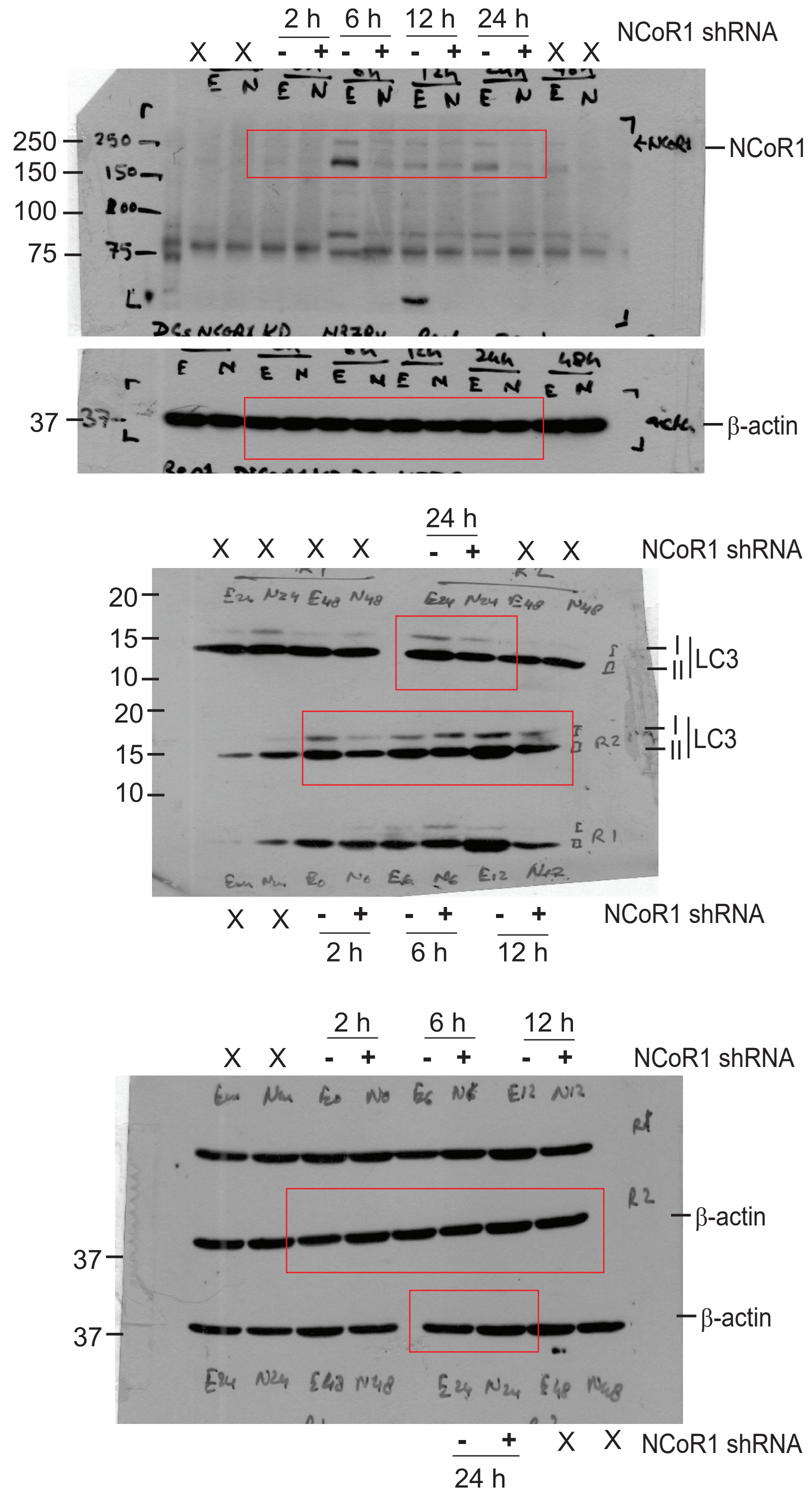

Supplementary Fig 4F

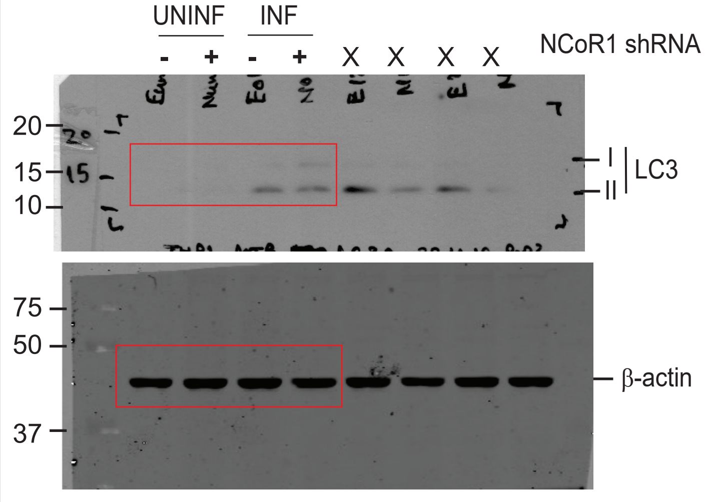

Supplementary Fig 5A

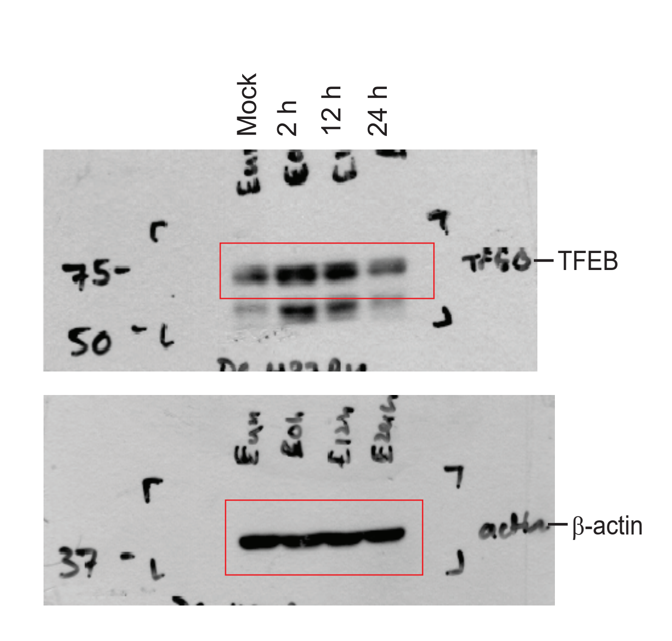

Supplementary Fig 5E

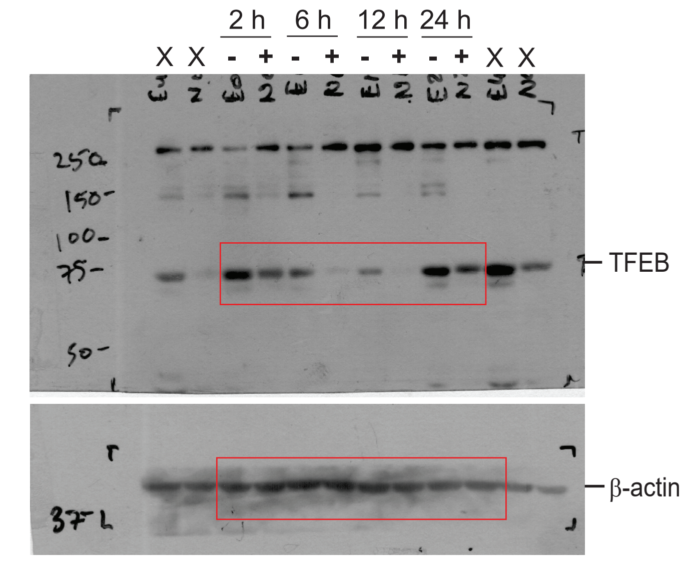

Supplementary Fig 5M

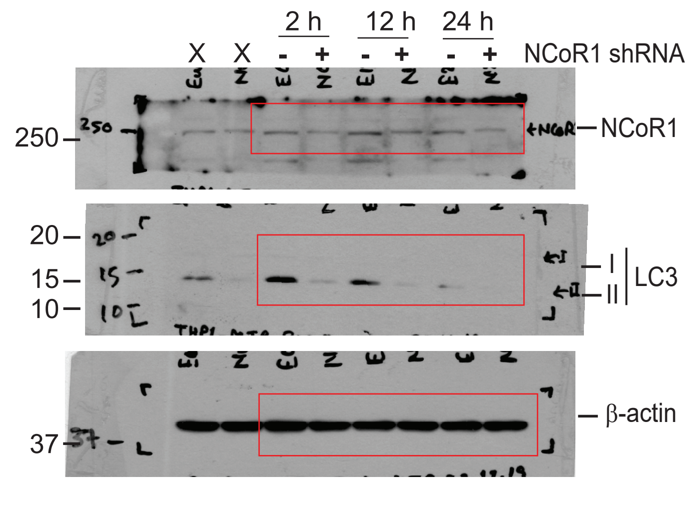

Supplementary Figure Western Blot Raw Images - 2

Supplementary Fig 5K

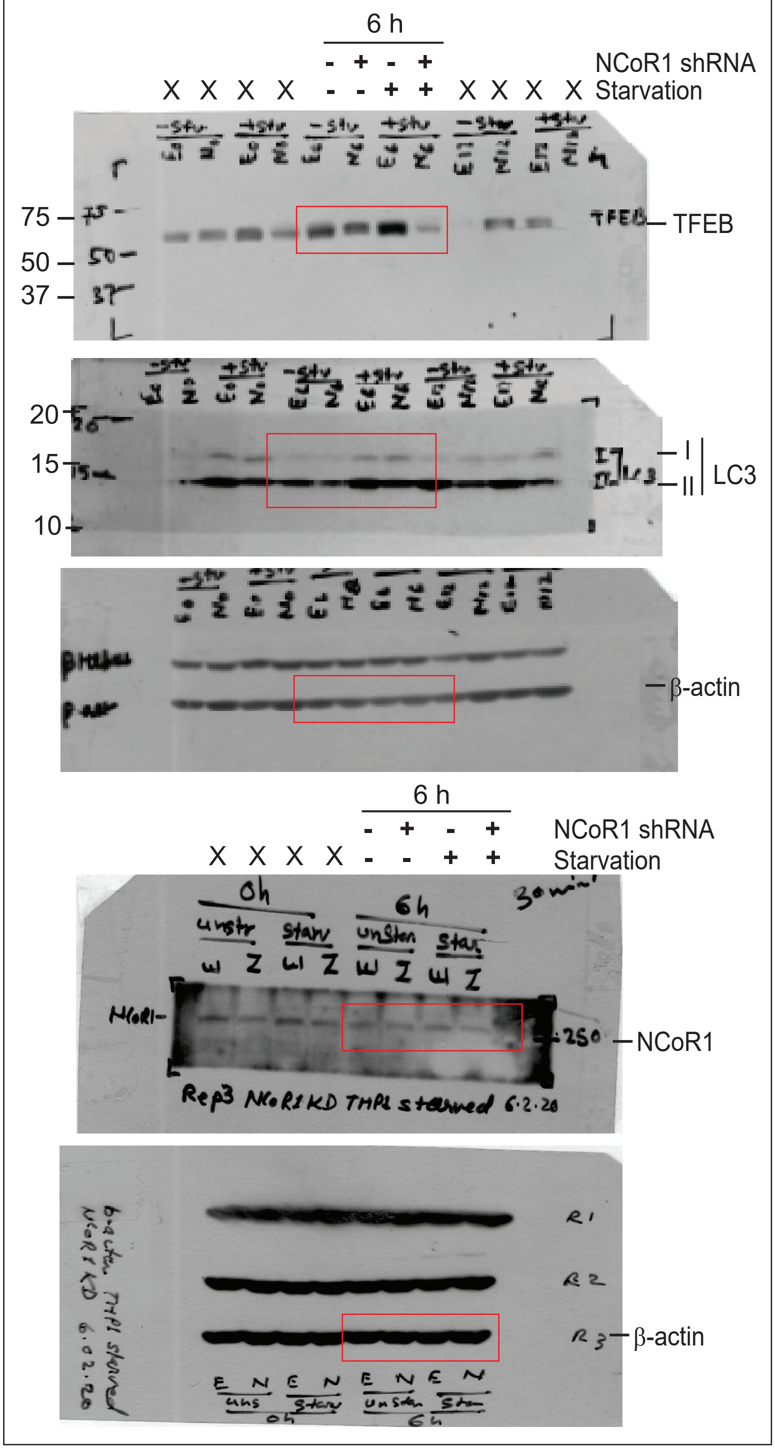

Supplementary Fig 5O

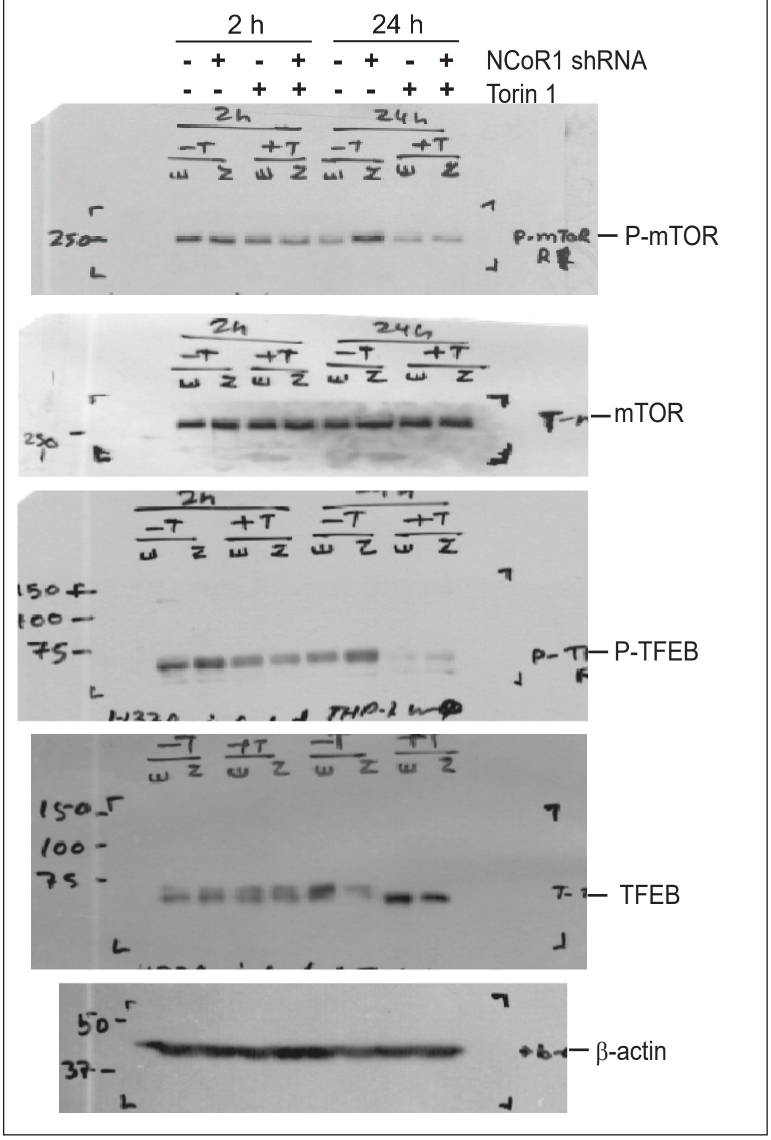

Supplementary Fig 6H

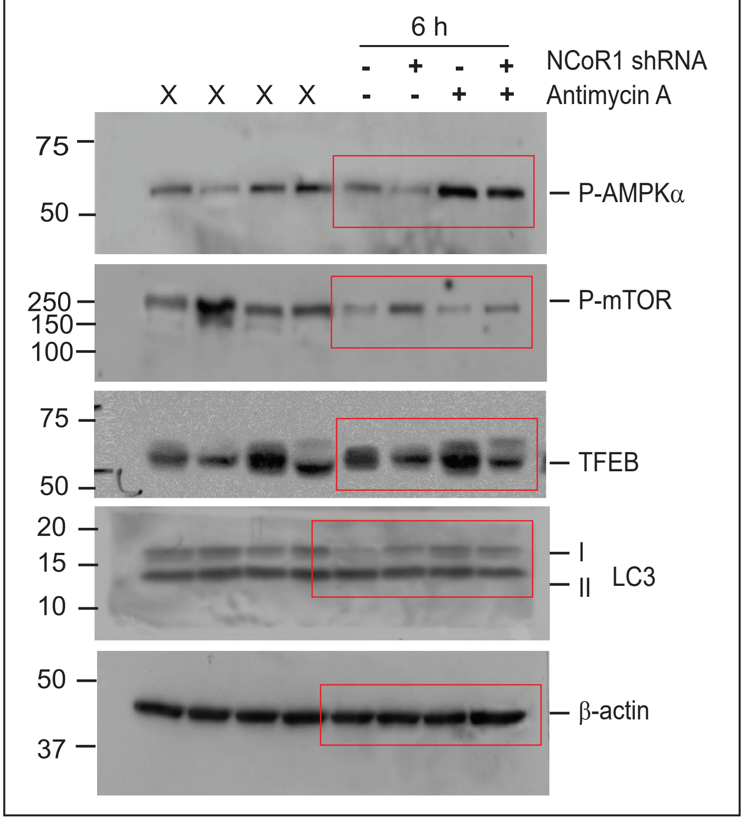

Supplement: S1 Raw Images — Figs 1F, 1G, 2A, 2I, 2N, 4D, 4G, 4I, 5A, 5B, 5D, 5E, 5H, 5J, 5L, 6B, 6H, 6K, S4D, S4F, S5A, S5E, S5M, S5K, S5O, and S6H. (PDF) [file pbio.3002231.s006.pdf]
