# Supplementary figures and images for: NCoR1 controls Mycobacterium tuberculosis growth in myeloid cells by regulating the AMPK-mTOR-TFEB axis
Source: PLoS Biol. 2023 Aug 17;21(8):e3002231. doi: 10.1371/journal.pbio.3002231 (PMC10465006; doi:10.1371/journal.pbio.3002231)

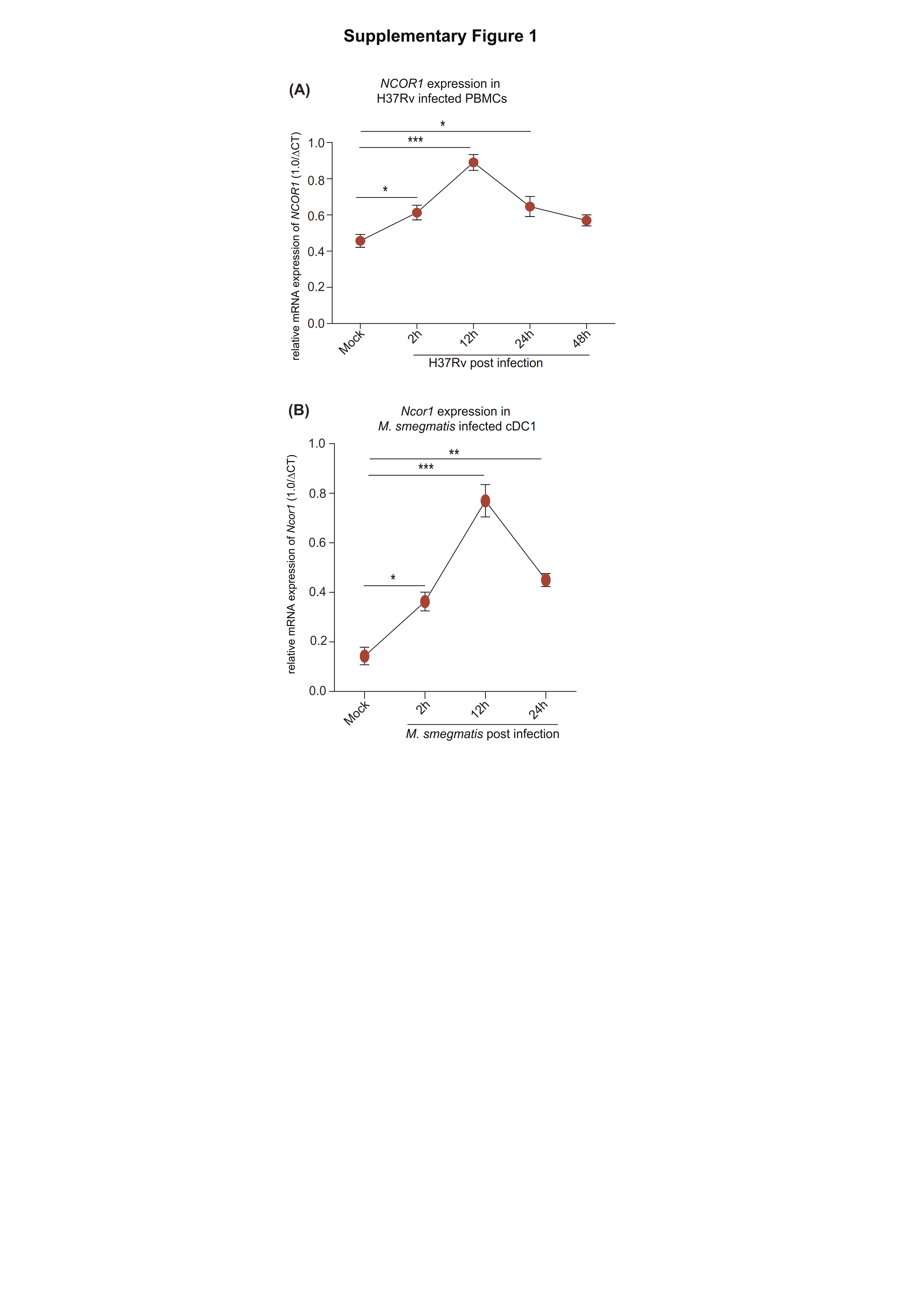

Supplement: S1 Fig — (A) RT-qPCR line graph showing the NCOR1 transcript kinetics (2 h, 12 h, 24 h, and 48 h) in H37Rv infected human PBMCs (n = 3 independent biological repeats). (B) RT-qPCR line graph showing the Ncor1 transcript kinetics (2 h, 12 h, and 24 h) upon M. smegmatis infection in cDC1 (n = 3 independent biological repeats). *p < 0.05, *p < 0.01, and ***p < 0.001 were considered significant. Data analysis was performed using one-way ANOVA with Tukey’s statistical test. Where n represents independent biological replicates. The data underlying this figure are available in S4 Table and S1 Data. (TIF) [file pbio.3002231.s007.tif]

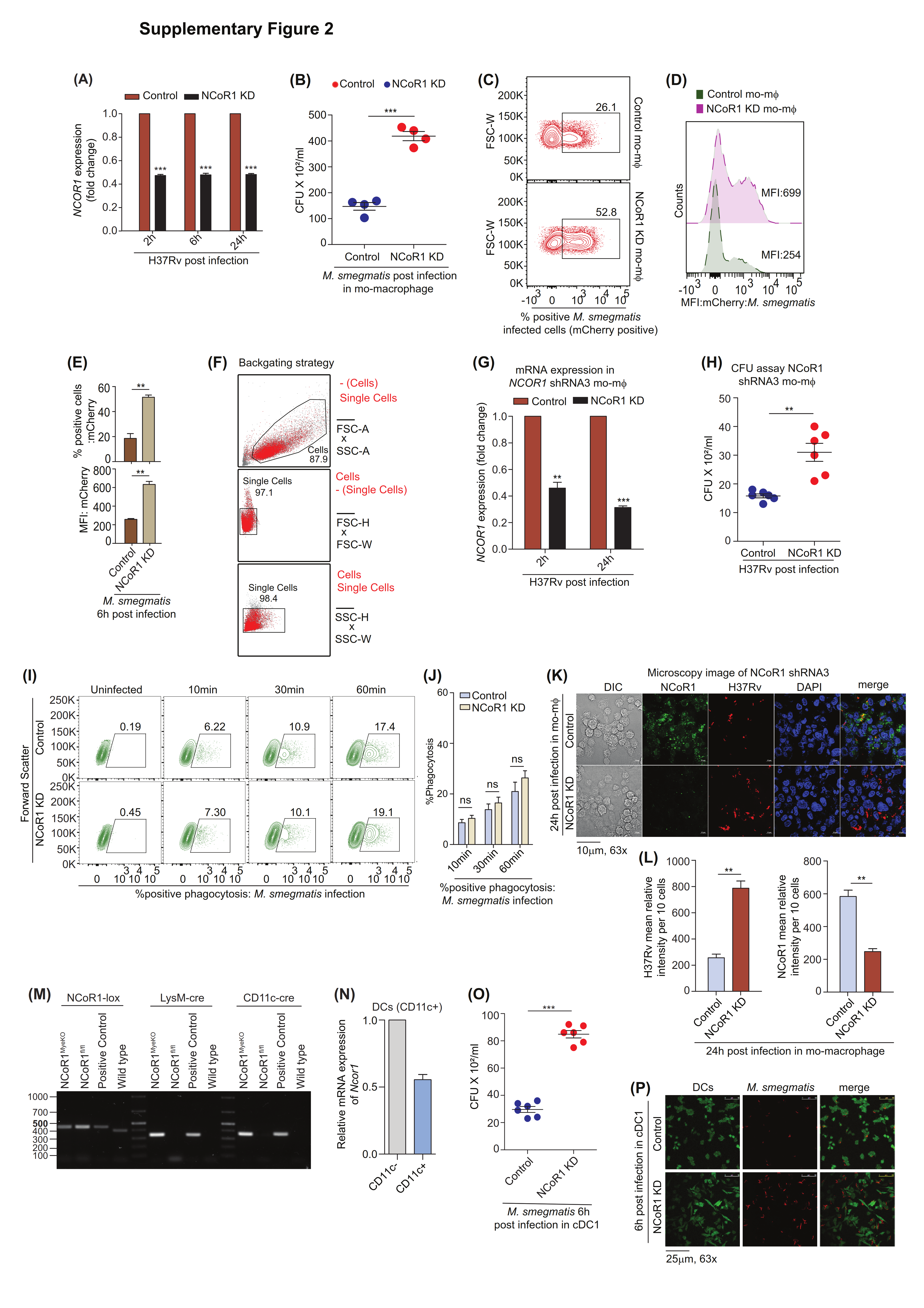

Supplement: S2 Fig — (A) Bar plot depicting the NCOR1 transcript expression at 2 h, 6 h, and 24 h of H37Rv infected control and NCoR1 KD human monocytic THP-1 differentiated macrophages (n = 3). (B) Scatter plot showing the M. smegmatis load in control and NCoR1 KD human monocytic THP-1 differentiated macrophages by CFU assay at 24 h post infection (n = 4). (C) Flow cytometry dot plot showing the percentage of M. smegmatis infected control and NCoR1 KD human monocytic THP-1 differentiated macrophages at 6 h post infection (n = 3). (D) Flow cytometry histograms showing MFI shifts for the M. smegmatis infection in control and NCoR1 KD human monocytic THP-1 differentiated macrophages at 6 h post infection (n = 3). (E) Bar plot showing the quantification of percent positive cells and MFI shifts for the M. smegmatis infected control and NCoR1 KD human monocytic THP-1 differentiated macrophages at 6 h post infection (n = 3). (F) Flow cytometry plots showing the back gating strategies used in flow cytometry analysis. (G) Bar plot of shRNA3-mediated NCoR1 depletion shown by RT-qPCR (n = 3). (H) Scatter plot showing the H37Rv load in control and shRNA3-mediated NCoR1 KD human monocytic THP-1 differentiated macrophages by CFU assay at 24 h (n = 6). (I) Flow cytometry contour plot showing the phagocytosis rate of GFP-tagged M. smegmatis in control and NCoR1 KD human monocytic THP-1 differentiated macrophages at 10 min, 30 min, and 60 min post infection (n = 3). (J) Bar plot showing the quantification of phagocytosis rate of M. smegmatis in control and NCoR1 KD human monocytic THP-1 differentiated macrophages at 10 min, 30 min, and 60 min post infection (n = 3). (K, L) Microscopy images and bar plot showing the levels of H37Rv infection in control and shRNA3-mediated NCoR1 KD at 24 h post infection (n = 3). (M) PCR results showing genotyping of NCoR1fl/fl and NCoR1MyeKO mice. (N) RT-qPCR depicting transcript levels of NCoR1 in CD11c+ cells compared to CD11c- fraction (n = 2 mice). (O) Scatter plot [file pbio.3002231.s008.tif]

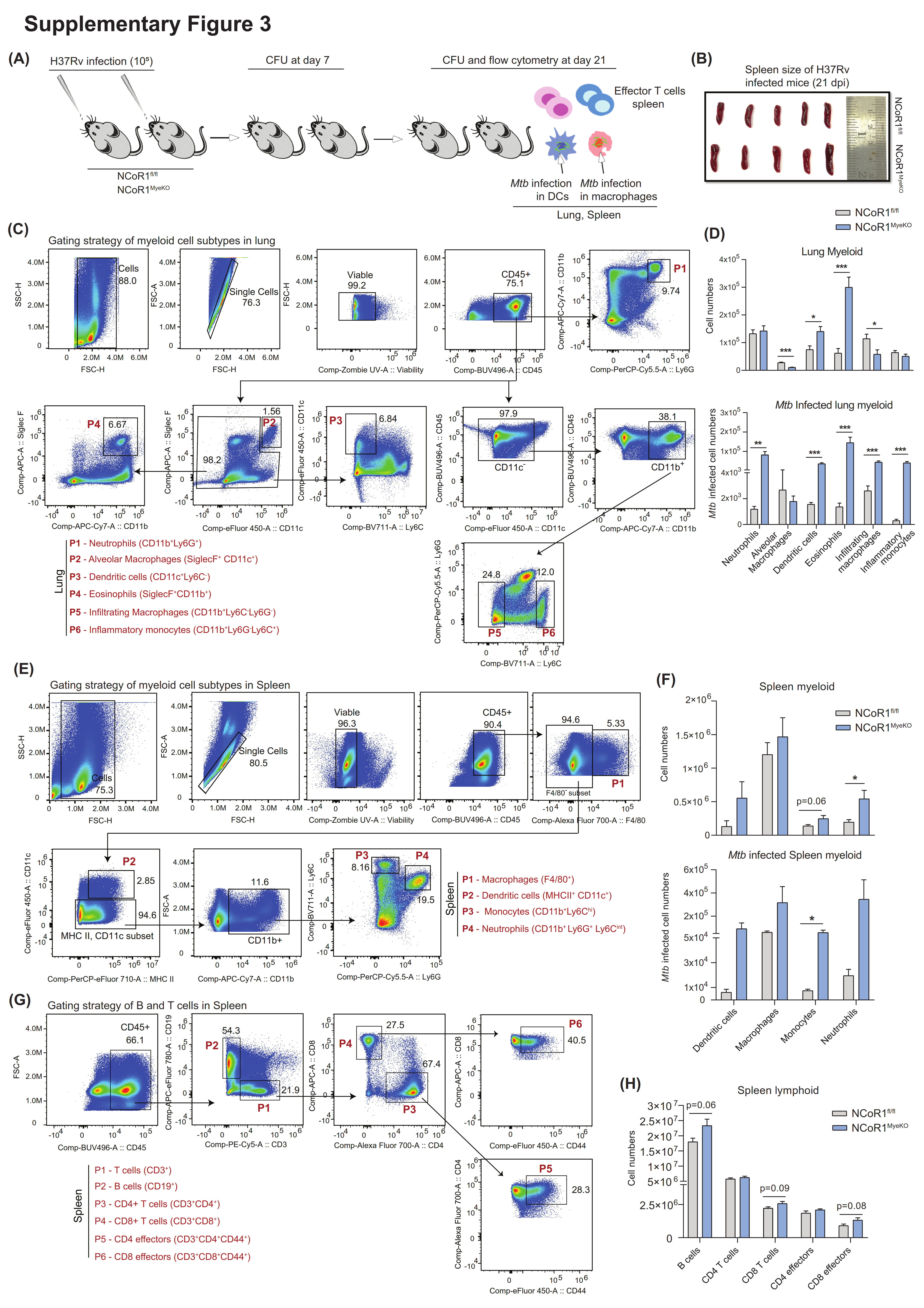

Supplement: S3 Fig — (A) Schematic outline depicting the in vivo experimental strategy for Mtb infection in mice. (B) Images showing the size of spleens isolated from NCoR1fl/fl and NCoR1MyeKO mice at 21 days post H37Rv infection (n = 5 mice). (C) Flow cytometry plots showing the gating strategy used for identification of myeloid cell subtypes in the lung tissues of NCoR1fl/fl and NCoR1MyeKO mice at day 21 post infection. (D) Bar plots depicting the myeloid cell numbers in the lung along with Mtb infected ones. (E) Flow cytometry plots showing the gating strategy used to analyse myeloid cell subtypes in the spleen tissues of NCoR1fl/fl and NCoR1MyeKO mice at day 21 post infection. (F) Bar plots demonstrating the myeloid cell numbers in the spleen along with Mtb infected ones. (G) Flow cytometry plots showing the gating strategy used to analyse B and T cell subtypes in the splenic tissues of NCoR1fl/fl and NCoR1MyeKO mice at day 21 post infection. (H) Bar plots showing the lymphoid cell numbers in the spleen. *p < 0.05, *p < 0.01, and ***p < 0.001 using unpaired two-tailed Student’s t test. Where n represents the total number of used mice. The data underlying this figure are available in S1 Data. (TIF) [file pbio.3002231.s009.tif]

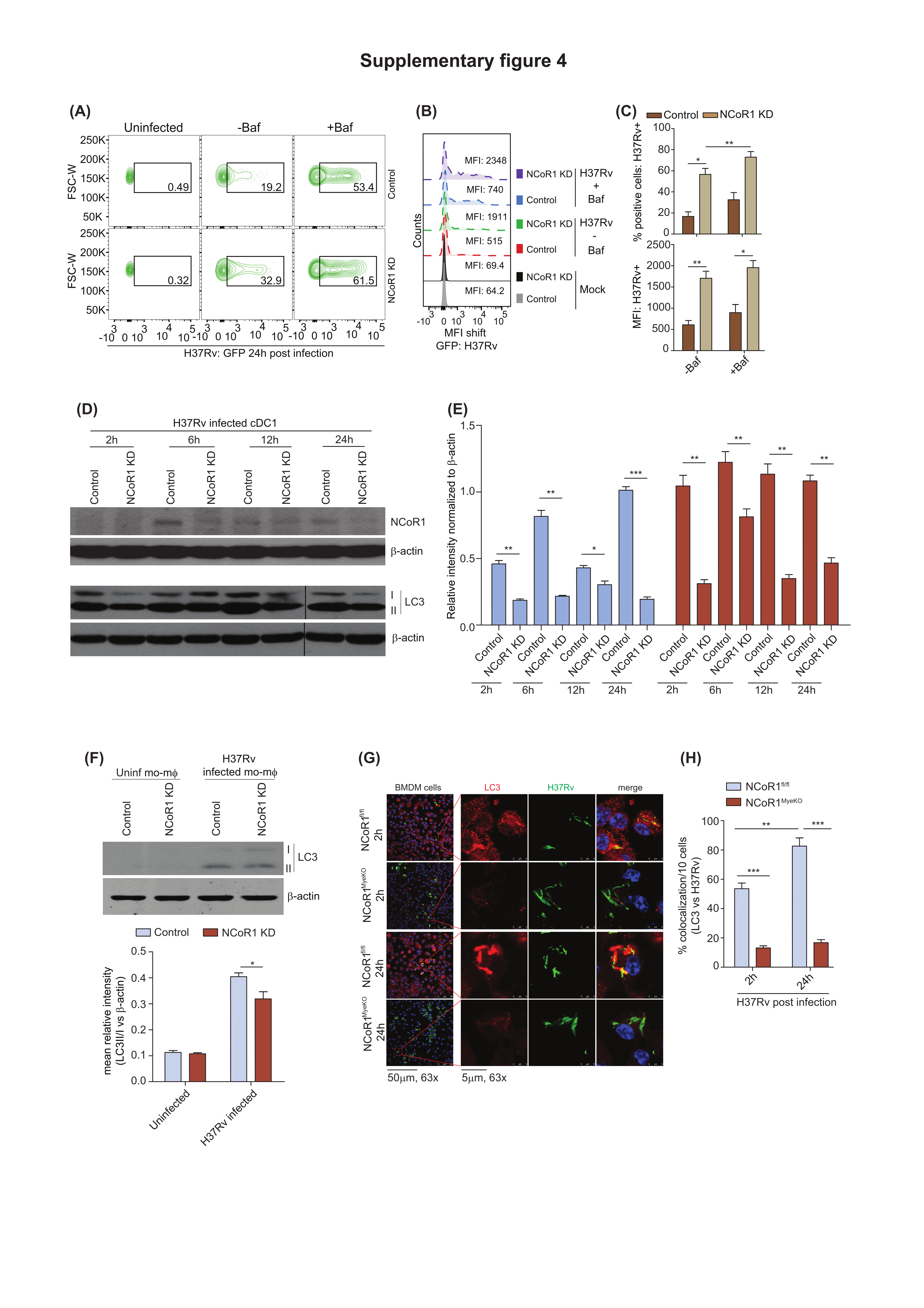

Supplement: S4 Fig — (A) Flow cytometry contour plots showing the intracellular H37Rv bacterial load in control and NCoR1 KD human monocytic THP-1 differentiated macrophages, with and without bafilomycin treatment (n = 3). (B, C) Flow cytometry histograms showing the MFI shifts for the H37Rv infection in control and NCoR1 KD human monocytic THP-1 differentiated macrophages with and without treatment of bafilomycin, bar plots depicting the quantification of the same (n = 3). (D) Western blot image showing the NCoR1 and LC3-II:LC3-I protein levels in control and NCoR1 KD cDC1 at different time points upon H37Rv infection (n = 3). (E) Bar plot showing densitometric quantification for the NCoR1 and LC3-II:LC3-I levels in control and NCoR1 KD cDC1 at different time points upon H37Rv infection. All protein bands were normalised with β-actin housekeeping control (n = 3). (F) Western blot image and corresponding densitometric analysis demonstrating the LC3-II:LC3-I protein levels in control and NCoR1 KD THP-1 differentiated mo-mΦ upon H37Rv infection vs. uninfected (n = 3). (G, H). Confocal microscopy and corresponding bar plot demonstrating the colocalization of H37Rv with LC3 protein in the BMDMs from NCoR1fl/fl and NCoR1MyeKO mice at 2 h and 24 h post infection (n = 4 mice). *p < 0.05, *p < 0.01, and ***p < 0.001 using paired and unpaired two-tailed Student’s t test, where n represents independent biological replicates. The data underlying this figure are available in S1 Data. Western blot raw images can be found in S1 Raw Image. (TIF) [file pbio.3002231.s010.tif]

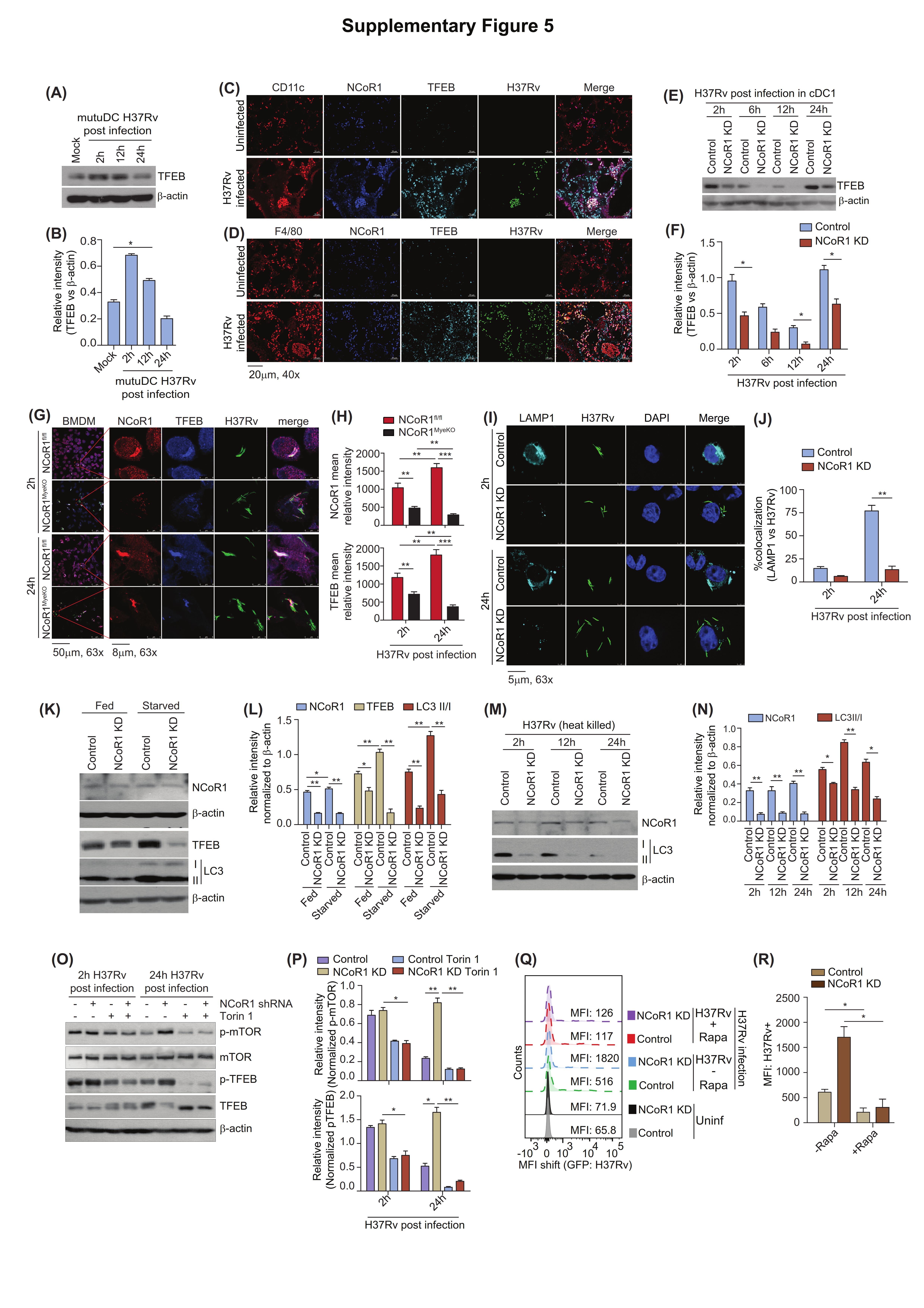

Supplement: S5 Fig — (A, B) Representative western blot image with corresponding densitometric analysis depicting the TFEB protein kinetics (2 h, 12 h, and 24 h) in the H37Rv infected cDC1. All protein bands were normalised with β-actin housekeeping control (n = 3). (C, D) Microscopy images showing the relative levels of NCoR1 and TFEB levels in CD11c+ and F4/80+ H37Rv-GFP infected lung tissue sections compared to uninfected C57BL/6 mice. (E, F) Western blot image and bar plot demonstrating the TFEB level in control and NCoR1 KD cDC1 at different time points upon H37Rv infection (n = 3). (G) Confocal microscopy showing NCoR1 and TFEB expression in H37Rv infected BMDMs generated from NCoR1MyeKO and NCoR1fl/fl mice (n = 4 mice). (H) Bar plot showing the quantification for NCoR1 and TFEB protein levels from confocal microscopy of H37Rv infected BMDMs generated from NCoR1MyeKO and NCoR1fl/fl mice (n = 4 mice). (I, J) Confocal microscopy images and bar plots showing the entrapment of H37Rv with LAMP1 protein in control and NCoR1 KD human monocytic THP1 differentiated macrophages at different time points (n = 3). (K) Western blot image showing the protein levels of NCoR1, TFEB, and LC3-II:LC3-I in starved and fed condition in control and NCoR1 KD human monocytic THP-1 differentiated macrophages (n = 3). (L) Bar plot showing densitometric quantification of NCoR1, TFEB, and LC3-II:LC3-I western bands in starved and fed condition in control and NCoR1 KD human monocytic THP-1 differentiated macrophages. All bands were normalised with β-actin as housekeeping control (n = 3). (M) Western blot image showing NCoR1 and LC3-II:LC3-I protein levels in control and NCoR1 KD human monocytic THP-1 differentiated macrophages treated with heat killed H37Rv at different time points (n = 3). (N) Bar plot depicting densitometric quantification of NCoR1 and LC3-II:LC3-I in control and NCoR1 KD human monocytic THP-1 differentiated macrophages treated with heat killed H37Rv at different time points. All bands were [file pbio.3002231.s011.tif]

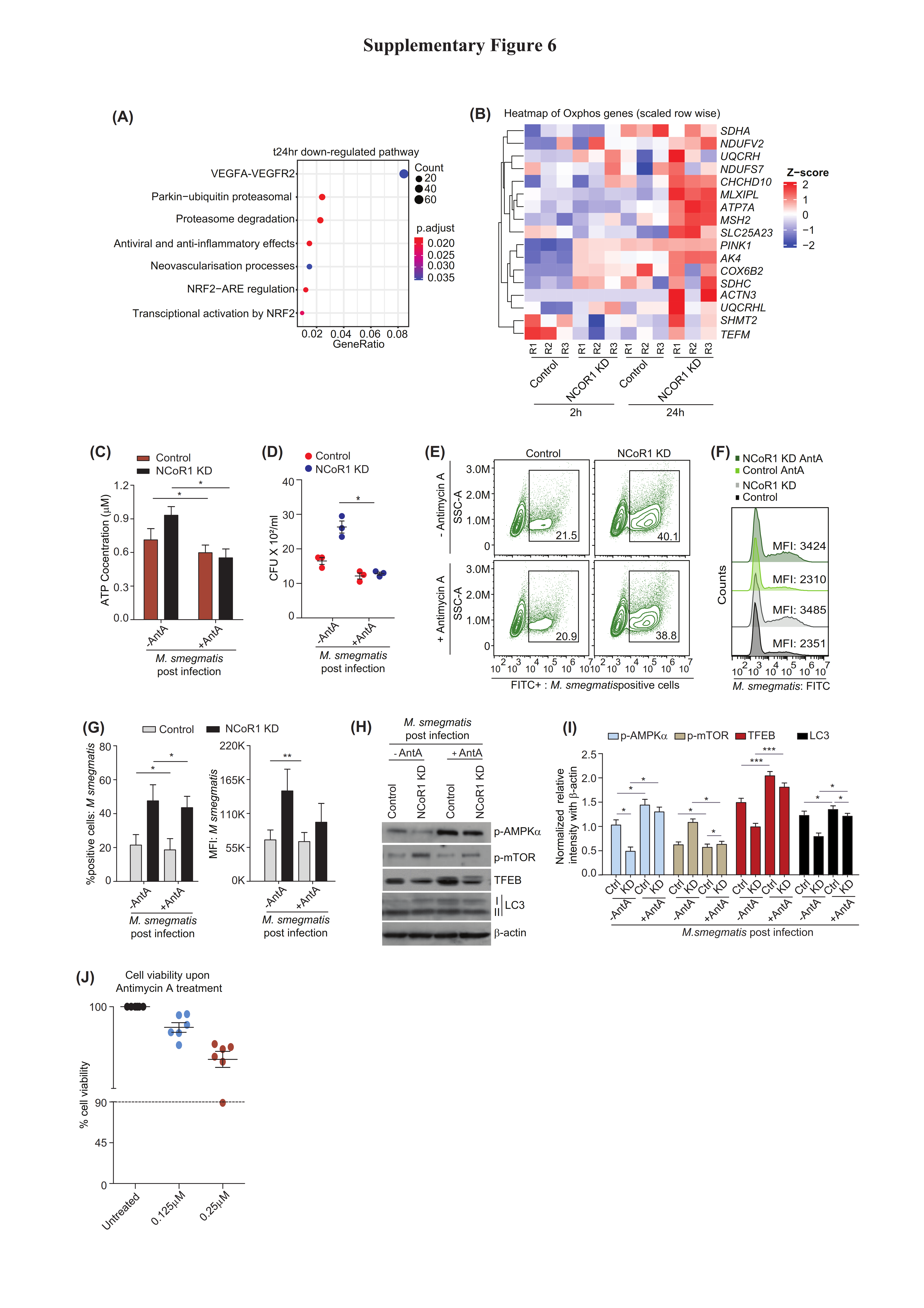

Supplement: S6 Fig — (A) Pathway enrichment analysis showing the top pathways for the list of down-regulated genes found in RNA-seq data of NCoR1 KD human monocytic THP-1 differentiated macrophages as compared to control cells at 24 h post H37Rv infection (n = 3). (B) Heat map depicting differentially expressed genes of oxidative phosphorylation in post-infected Control and NCoR1 KD cells (n = 3). (C) Bar graph showing the intracellular ATP level in control and NCoR1 KD human monocytic THP-1 differentiated macrophages upon M. smegmatis infection at 6 h time point with and without antimycin A treatment (n = 3). (D) Scatter plot demonstrating the M. smegmatis bacterial load in control and NCoR1 KD human monocytic THP-1 differentiated macrophages at 6 h time point by CFU assay with and without antimycin A treatment (n = 3). (E) Flow cytometry contour plots depicting the GFP-tagged M. smegmatis infection in control and NCoR1 KD human monocytic THP-1 differentiated macrophages at 6 h with and without antimycin A treatment (n = 3). (F) Flow cytometry histogram plots showing the MFI shifts for M. smegmatis infection load in control and NCoR1 KD human monocytic THP-1 differentiated macrophages at 6 h with and without antimycin A treatment (n = 3). (G) Bar plots depicting the quantitation of percent positive infected cells and corresponding MFI shifts in flow cytometry analysis of M. smegmatis infection in control and NCoR1 KD human monocytic THP-1 differentiated macrophages at 6 h with and without antimycin A treatment (n = 3). (H) Western blot representative image demonstrating the p-AMPKα, p-mTOR, TFEB, and LC3 protein levels in M. smegmatis infected control and NCoR1 KD human monocytic THP-1 differentiated macrophages with and without antimycin A treatment (n = 3). (I) Bar plots demonstrating the quantification of p-AMPKα, p-mTOR, TFEB, and LC3 western blot bands in M. smegmatis infected control and NCoR1 KD human monocytic THP-1 differentiated macrophages with and without antimycin A treatm [file pbio.3002231.s012.tif]
