# Supplementary material for: NCoR1 controls Mycobacterium tuberculosis growth in myeloid cells by regulating the AMPK-mTOR-TFEB axis
Source: PLoS Biol. 2023 Aug 17;21(8):e3002231. doi: 10.1371/journal.pbio.3002231 (PMC10465006; doi:10.1371/journal.pbio.3002231)
Supplement: S7 Fig — “Created with BioRender.com.” (PDF) [file pbio.3002231.s013.pdf]

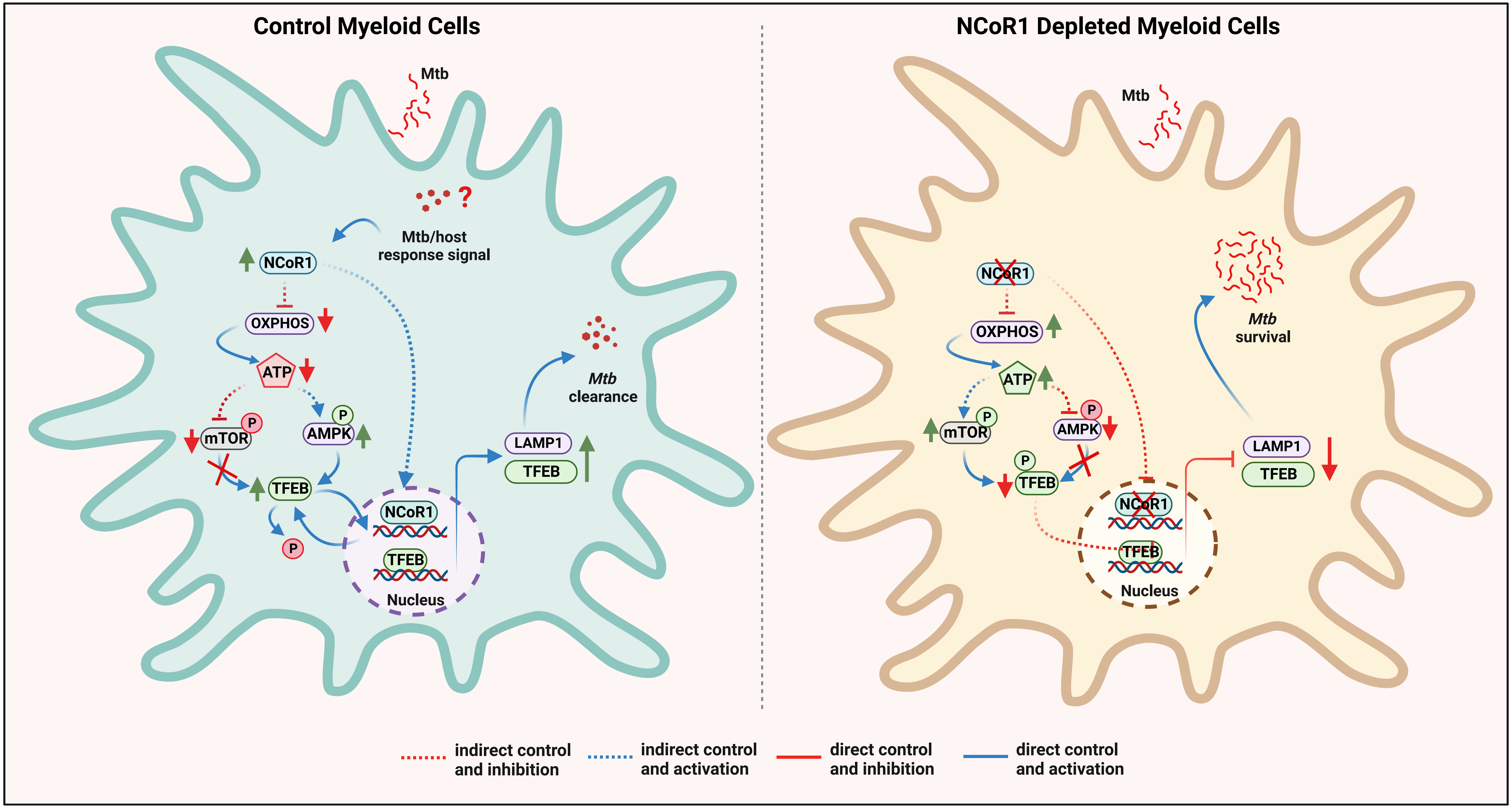

Control Myeloid Cells
NCoR1 Depleted Myeloid Cells
Mtb
Mtb/host
response signal
NCoR1
OXPHOS
ATP
mTOR
AMPK
P
TFEB
LAMP1
Mtb
clearance
Nucleus
Mtb
survival
indirect control
and inhibition
indirect control
and activation
direct control
and inhibition
direct control
and activation
